# Mycobacterial Populations Partly Change the Proportions of the Cells Undergoing Asymmetric/Symmetric Divisions in Response to Glycerol Levels in Growth Medium

**DOI:** 10.3390/cells10051160

**Published:** 2021-05-11

**Authors:** Atul Pradhan, Nagaraja Mukkayyan, Kishor Jakkala, Parthasarathi Ajitkumar

**Affiliations:** Department of Microbiology and Cell Biology, Indian Institute of Science, Bangalore 560012, Karnataka, India; atul0276@gmail.com (A.P.); nag.864@gmail.com (N.M.); kishorchembio@gmail.com (K.J.)

**Keywords:** asymmetric constriction during division, symmetric constriction during division, asymmetric/symmetric division switching, glycerol levels, *Mycobacterium smegmatis*, gene knockout mutants

## Abstract

Twenty to thirty percent of the septating mycobacterial cells of the mid-log phase population showed highly deviated asymmetric constriction during division (ACD), while the remaining underwent symmetric constriction during division (SCD). The ACD produced short-sized cells (SCs) and normal/long-sized cells (NCs) as the sister–daughter cells, but with significant differential susceptibility to antibiotic/oxidative/nitrite stress. Here we report that, at 0.2% glycerol, formulated in the Middlebrook 7H9 medium, a significantly high proportion of the cells were divided by SCD. When the glycerol concentration decreased to 0.1% due to cell-growth/division, the ACD proportion gradually increased until the ACD:SCD ratio reached ~50:50. With further decrease in the glycerol levels, the SCD proportion increased with concomitant decrease in the ACD proportion. Maintenance of glycerol at 0.1%, through replenishment, held the ACD:SCD proportion at ~50:50. Transfer of the cells from one culture with a specific glycerol level to the supernatant from another culture, with a different glycerol level, made the cells change the ACD:SCD proportion to that of the culture from which the supernatant was taken. RT-qPCR data showed the possibility of diadenosine tetraphosphate phosphorylase (*MSMEG_2932*), phosphatidylinositol synthase (*MSMEG_2933*), and a Nudix family hydrolase (*MSMEG_2936*) involved in the ACD:SCD proportion-change in response to glycerol levels. We also discussed its physiological significance.

## 1. Introduction

Bacterial populations are known to maintain phenotypic heterogeneity in cell-length, cell size, morphology, cellular components, metabolic status, and in several other factors, for survival under diverse growth and stress conditions [1,2]. Both pathogenic and non-pathogenic mycobacteria maintain phenotypic heterogeneity in in vitro cultures, mice, guinea pigs, and tuberculosis patients [3,4,5,6,7,8,9,10]. Mycobacterial phenotypic heterogeneity, in terms of cell-size and morphology, has been found to correlate with differential tolerances to stress conditions, such as nutrient depletion [3,11,12,13,14,15,16,17], antibiotics [18,19,20,21,22,23], and others. Thus, phenotypic heterogeneity among subpopulations has a deeper implication, in terms of metabolic differences in mycobacteria and their beneficial outcome in the tolerance to diverse types of stress conditions.

Among the diverse phenotypes, cell-length heterogeneity can be achieved by generating differently sized sister–daughter cells. Many bacterial genera, including mycobacteria, divide asymmetrically to produce differently sized sister–daughter cells. The different means of generating differently sized sister–daughter cells in the mycobacterial population include budding of short-sized daughter cells from the cell-tips of multi-septated *Mycobacterium tuberculosis* long-sized mother cells [24]. The differential rates in elongation of sister–daughter cells in a dividing mother cell in *Msm* is also found to be a reason behind heterogeneity in sister–daughter cell lengths [25]. In this regard, we found that 20–30% of the septating mid-log phase (MLP) cells of *Mycobacterium tuberculosis*, *Mycobacterium smegmatis*, and *Mycobacterium xenopi* populations undergo highly deviated asymmetric constriction during division (ACD; 11–30% from the median) [9,10]. Thus, ACD generated short-sized cells (SCs) and normal/long-sized cells (NCs) as sister–daughter cells. The remaining 70–80% of the septating cells undergo symmetric constriction during division (SCD), with only 5–10% deviation from the median [9,10,26]. The SCs and the NCs would undergo either ACD or SCD to generate more SCs and NCs [21]. The difference in cell-length was found to have correlation with difference in cell density, with the SCs having low buoyant density than the NCs. This difference in the buoyant density in turn was reflective of the differences in their metabolic status that conferred differential susceptibility to antibiotics/oxidative/nitrite stress conditions [21,22,23].

Earlier studies reported that late growth phases of *M. smegmatis*, *Mycobacterium bovis* BCG, and *M. tuberculosis* cells contained short-sized cells [5,6], which were suggested to be formed due to reductive cell division [5]. While cell-size reduction by reductive division in response to nutritional decline is a possibility, growth phase specific changes in the proportions of mycobacterial cells dividing by ACD and SCD, in response to nutritional status, also might give rise to short/long-sized sister–daughter cells. Therefore, we wanted to verify the possibility as to whether mycobacterial cells would alter the proportions of the cells dividing by ACD and SCD during different growth phases vis-à-vis glycerol levels in Middlebrook 7H9 medium and identify specific genes influencing the change in the proportions of the cells dividing by ACD/SCD. We focused only on the influence of glycerol, and not on other possible carbon sources, as glycerol was found to be the most ideal carbon source for the efficient growth and metabolism of mycobacteria under in vitro conditions [27].

## 2. Materials and Methods

### 2.1. Bacterial Strains and Culture Conditions

*Mycobacterium smegmatis* mc^2^155 (*Msm*) cells [28] were cultured in Middlebrook 7H9 medium (Difco; BD Bioscience, Franklin Lakes, NJ, USA) containing 0.2% glycerol (Sigma-Aldrich, Saint Louis, MO, USA) and 0.05% Tween 80 (Sigma-Aldrich, Saint Louis, MO, USA), as stipulated in the formulation of the medium [27]. Primary culture was prepared fresh, each time, in triplicate, from the same fresh glycerol stock by inoculating 200 μL of the glycerol stock into 20 mL of the medium, in a 100 mL flask, and incubated at 37 °C, 170 rpm, until the culture reached mid-log phase (optical density at 600 nm, OD_600 nm_, of 0.6). Since OD values were always measured at 600 nm, OD_600 nm_ is mentioned as OD throughout the manuscript. Clump-free secondary cultures, in triplicate, with comparable OD values for experiments were initiated with 1% inoculum from the triplicate primary cultures. *Escherichia coli* strains for plasmid propagation were cultured in Luria–Bertani (LB) medium (BD Bioscience, Franklin Lakes, NJ, USA) [29,30] (Supplementary Table S1).

### 2.2. Growth Curve Construction

One percent inoculum from the 0.6 OD_600 nm_ primary culture, which started from the glycerol stock of *Msm*, was added to 100 mL of Middlebrook 7H9 medium, containing 0.2% glycerol and 0.05% Tween 80. The OD_600 nm_ values at different time points were measured in UV-Visible spectrophotometer (Shimadzu UV-1800 UV/Visible scanning spectrophotometer, Kyoto, Japan), using one mL aliquots of the culture (BRAND^®^ semi-micro cuvette, Wertheim, Germany; minimum filling volume 1.5 mL), with fresh unused Middlebrook 7H9 growth medium as the blank. Data were analyzed with MS-Excel, 2013, for constructing growth curve. Generation time was calculated using the formula, mass doubling time = [0.693 × (t2 − t1) ÷ (log_10_ C2 − log_10_ C1)] × 2.303, where t1 and t2 are the time of initiation and end of log phase, respectively, and C1 and C2 are the ODs at the start and the end of log phase, respectively [31].

### 2.3. Estimation of Glycerol Concentration

The concentration of free glycerol was determined by its oxidation with potassium periodate under acidic condition to formaldehyde, along with formic acid, iodate, and water [32].

CH_2_(OH)·CH(OH)·CH_2_OH + 2IO_4_^−^ → 2H·CHO + H·CO_2_H + H_2_O + 2IO_3_^−^

The formaldehyde undergoes Hantzsch reaction with acetyl acetone, in the presence of ammonium salt, to form a yellow-colored compound, diacetyl dihydro lutidine (DDL), which can be colorimetrically monitored to determine the concentration of formaldehyde formed [33,34]. This conversion of glycerol to formaldehyde, and formaldehyde to DDL, was used to establish a protocol to find out the concentration of free glycerol, in biodiesel sample [35], and in bacterial and yeast fermentation media [36], by the spectrophotometric estimation of DDL at 410 nm. Free glycerol concentration in the culture supernatant was measured at different time points, as described [35], with minor modifications. In brief, the reagents were prepared as follows. (i) Acetic acid stock solution: 1.6 M aqueous solution; (ii) ammonium acetate stock solution: 4.0 M aqueous solution; (iii) 0.2 M acetyl acetone solution: 200 µL of acetyl acetone was added into 5 mL of acetic acid stock solution, mixed well, and 5 mL of ammonium acetate stock solution was added to it; (iv) 10 mM sodium (meta) periodate solution: 21 mg of sodium (meta) periodate (Sigma-Aldrich, Saint Louis, MO, USA) were dissolved in 5 mL of acetic acid stock solution, and once sodium (meta) periodate is completely dissolved, 5 mL of ammonium acetate stock solution was added to it. (v) Working solvent: equal volumes of autoclaved double distilled water and 95% ethanol (Merck, Darmstadt, Germany).

Different concentrations of glycerol standard were prepared as 0.4, 0.2, 0.1, 0.05, 0.025, 0.0125, and 0.00625% by serial dilution using autoclaved double distilled water. Thirteen µL of glycerol standard was dissolved in 117 µL of distilled water. Three hundred and ninety (390) µL of working solvent was added to 130 µL of the diluted standard. Three hundred and twelve (312) µL of sodium (meta) periodate solution was added and mixed on a vortex mixer for 30 secs. Subsequently, 312 µL of 0.2 M acetyl acetone (Sigma-Aldrich, Saint Louis, MO, USA) solution was added and kept in a heating block at 70 °C for 1 min. The sample was then immediately cooled by keeping the tube on ice. Absorbance was measured immediately at 410 nm in a UV-Visible spectrophotometer. Standard calibration curve was constructed and equation for the straight line, y = mx + c, was used to determine the glycerol concentration in the culture medium, where y is absorbance at 410 nm, m is the slope, c is the Y-axis intercept, and x is the unknown concentration of glycerol to be determined as x = (y − c) ÷ m.

The glycerol concentration in the culture supernatants was determined as follows. Culture supernatants from the cultures at different time points were collected by centrifuging one mL of *Msm* culture at ~5000× *g* for 10 min at 25 °C, from independently grown biological triplicate cultures. Samples were treated with sodium (meta) periodate and acetyl acetone solution in the same way as in the case of the standards. Absorbance of the DDL formed was measured at 410 nm. Culture supernatants at different time points without any inoculum and glycerol were taken as blank, for the respective time points. Glycerol concentration (X-axis intercept) was calculated with the equation of the straight line of the standard calibration curve, referred to above.

### 2.4. Measurement of Birth-Lengths of the Cells Dividing by ACD and SCD

*Msm* cells were harvested from secondary cultures, at different growth phases from 0.2 OD_600 nm_ at every 0.2 OD_600 nm_ interval, until stationary phase, by withdrawing 1 mL of well shaken culture and centrifuging at ~5000× *g* at 4 °C for 10 min. Cell pellets were fixed in 4% paraformaldehyde (*w/v*) (Merck, Darmstadt, Germany) prepared in 1x phosphate buffered saline (PBS) and then washed with 1x PBS after 1 h of incubation at 25 °C [9]. The cells were then adhered to poly-l-lysine (Sigma-Aldrich, Saint Louis, MO, USA) coated multi-well slides and DIC images were captured with Zeiss AXIO imager microscope (ZEISS, Oberkochen, Germany) and analyzed using ZEN software (Carl Zeiss ZEN2; Carl Zeiss Microscopy GmbH, Göttingen, Germany). Sister–daughter cell-lengths were measured for 300 dividing cells from each growth phase. Cell-lengths of dividing mother cells were measured from the site of constriction to the end of the poles and the difference was calculated [9,10,37]. The sister–daughter cells were named as daughter cell one (D1) and daughter cell two (D2) where D1 > D2 in length was kept throughout this study. The dividing cells were identified by a clear constriction visible in the DIC images. For the proportion of ACD and SCD in the manuscript, the *Msm* cells showing constriction or undergoing V-snapping after constriction [6,24], were considered for counting and measuring sister–daughter cell-lengths. For demarcation between the cells undergoing SCD and ACD, based on our earlier published observations [9,10,37], the mother cells, which were V-snapped after constriction, generating D1 and D2 differing in length by ≥11% deviation from mid-cell site, were considered as dividing by ACD. Similarly, D1 and D2 differing in length by ≤11% deviation from mid-cell site, were considered as dividing by SCD. The percent deviation of constriction from mid-cell site was calculated as (D1 − D2) ÷ (D1 + D2) × 100, using MS Excel 2013. The details of the procedures involved in cell harvesting all of the way up to data acquisition for calculating the proportion of ACD and SCD are presented in Scheme S1. The significance of the results was calculated using the two-tailed paired *t*-test, using GraphPad PRISM^®^ 5.01 software (GraphPad Software, San Diego, CA, USA).

### 2.5. Calculation and Statistical Analyses of the Frequency of ACD and SCD

The following parameters were calculated as described [38,39,40]: (i) the ratio P of the length of each sister–daughter cell to the length of the respective constricted mother cell (two values of P are obtained for every constricted cell); (ii) the probability density of P, called K(P), which shows symmetric or asymmetric distribution; and (iii) the coefficient of variation of P (CV; standard deviation ÷ mean), which is a measure of the extent of deviation of the division constriction from the median. Accordingly, the ratio P = length of each of the newly formed sister–daughter cell ÷ length of the constricted mother cell. The value of P will be distributed close to 0.5 for both the sister–daughter cells in SCD but will be far below (for one sister–daughter cell) or far above (for the other sister–daughter cell) 0.5 for the sister–daughter cells in ACD.

The percentages of cells undergoing ACD and SCD (on *Y*-axis) were plotted against the ratio P (on *X*-axis) for each constricting mother cell from every OD value (*n* = 300 in each case) to get the distribution of P. For SCD, the distribution of P will be unimodal and may show a single Gaussian peak. However, for ACD, the distribution of P will still be Gaussian for each sister–daughter cell, but will show up as bimodal (two peaks) on either side of 0.5 value of P. Thus, single peak distribution of dividing cell population will indicate SCD, and bimodal distribution of dividing cell population will indicate systematic ACD. Since the CV for the P measurements for both the sister–daughter cells from SCD at each OD value will be comparable, the CV% for the daughter cells from SCD will be of high precision and of low percentage. On the contrary, since the CV for the P measurements for both the daughter cells from ACD at each OD value will not be comparable, the CV% for the daughter cells from ACD will be of low precision and of higher percentage.

Geary’s statistics was applied to find out *w_n_*, which is the ratio of the mean deviation to the standard deviation, as a test of normality of distribution of the population [41]. If a population is normally distributed, the sample value of *w_n_* will be ~0.7979 when N → ∞. For the test of K(*P*) distributions, N is the number of dividing cells. Geary’s statistics *w_n_* was used to measure kurtosis (peakedness) of K(*L*) [40]. If variable P is considered as a mixture of two normally distributed random variables, say P D1 and P D2, then the K(*P*) will be platykurtic, with *w_n_* > 0.7979. A significant platykurtosis will be an indication of systematic asymmetry in the dividing cells.

### 2.6. Effect of Glycerol Replenishment on ACD and SCD Proportions

By measuring OD at 3 h interval, the growth curve was plotted for *Msm* cells cultured in 100 mL of Middlebrook 7H9 medium containing 0.2% glycerol and 0.05% Tween 80. Glycerol concentration in the culture supernatants from every time point/OD was determined, as described earlier. When the glycerol concentration in the culture supernatant reached ~0.1%, autoclaved glycerol was added exogenously to a final concentration of 0.05% at 16, 19 and 22 h to maintain the glycerol levels at ~0.1%. Cells were harvested and fixed with 4% paraformaldehyde (PFA) (*w/v*) prepared in 1x PBS, as described earlier. Cell-length of constricted mother cells was measured using DIC images of fixed cells, as described earlier, to find out the proportion of the cells dividing by ACD and SCD in the glycerol-replenished culture. *Msm* culture without exogenous addition of glycerol was used as the control. Similarly, the same quantity of Middlebrook 7H9 media (50 µL) was added at 16, 19, and 22 h, as performed for exogenous glycerol, at 1X and 10X concentration of Middlebrook 7H9, keeping the Tween 80 same at 0.05%, in individual cultures, to see the effect of Middlebrook 7H9 media instead of glycerol. Significance of the results was calculated using the two-tailed paired *t*-test and the two-tailed unpaired *t*-test, using GraphPad PRISM^®^ 5.01 software.

### 2.7. Culturing Msm Cells in Middlebrook 7H9 Medium Containing 0.1% Glycerol and 0.05% Tween 80

One percent of 0.6 OD culture, in Middlebrook 7H9 medium containing 0.2% glycerol and 0.05% Tween 80, generated from glycerol stock was inoculated into Middlebrook 7H9 growth medium containing 0.1% glycerol and 0.05% Tween 80. The cells in the inoculum were not washed free of glycerol since 1% inoculum (i.e., 1 mL in 100 mL culture) will give only 0.003% additional glycerol in 100 mL medium. One mL aliquots of the *Msm* culture were withdrawn at different time points (3 to 24 h, at 3 h interval) and OD was measured using fresh Middlebrook 7H9 medium without the cells as the blank. Growth curve was plotted, and generation time was calculated as mentioned earlier. Glycerol concentration in the medium was also determined, as described above. Harvested cells were fixed in 4% PFA (*w/v*) prepared in 1x PBS and then washed with 1x PBS after 1 h incubation at 25 °C. DIC images were taken as described earlier and analyzed for the proportion of the cells constricting by ACD and SCD, as described above. Significance of the results was calculated using the two-tailed paired *t*-test, using GraphPad PRISM^®^ 5.01 software.

### 2.8. Effect of Culture Supernatants of Different OD on ACD/SCD Proportions

The cells from different *Msm* cultures at 0.2, 1.2, and 2.0 OD, were centrifuged at ~5000× *g* at 4 °C for 10 min. The culture supernatants were aseptically collected and filtered through 0.22 µm filter (Millipore, Bangalore, India) to remove the cells. Hundred (100) µL of culture supernatant was plated on 7H10 agar (BD GmbH, Heidelberg, Germany) containing 0.2% glycerol and incubated at 37 °C for 48 h, to confirm the absence of cells in the supernatant. The cells from the cultures having different OD were resuspended in the culture supernatants from other OD cultures and incubated by shaking at 170 rpm, 37 °C. The OD values and glycerol concentrations were measured at 0 h, 3 h, 6 h, and 9 h post-suspension of the cells in the culture supernatant. Cells were collected by centrifugation at ~5000× *g* for 10 min at 4 °C, fixed with 4% PFA (*w/v*) prepared in 1x PBS, and then washed with 1x PBS after 1 h incubation at 25 °C. DIC images were taken and quantitated for the proportion of the cells dividing by ACD and SCD, as described earlier. Significance of the results was calculated using the two-tailed paired *t*-test, using GraphPad PRISM^®^ 5.01 software.

### 2.9. Estimation of ACD/SCD Proportions in the Ap_6_A Exposed Msm MLP Cells

*Msm* cells (secondary culture), growing in 50 mL Middlebrook 7H9 medium at 0.6 OD, were harvested at ~5000× *g* for 10 min at RT. The cells were transferred to 10 mL fresh Middlebrook 7H9 medium, in 50 mL flask and treated with 166 pM synthetic Ap_6_A (Jena Bioscience, Jena, Germany) [37] for 1 h, at 37 °C, 170 rpm. After 1 h, 200 µL of the cells was harvested and fixed with 4% PFA, as described earlier, for the determination of ACD:SCD proportions. The remaining cells from the 10 mL culture were harvested at ~5000× *g* for 10 min at 4 °C and snap-frozen in liquid nitrogen and stored at −75 °C for RNA extraction. The cells from the MLP and 0.8 OD cultures (unexposed to Ap_6_A), in 50 mL culture in 250 mL flask, were also harvested for the determination of ACD/SCD proportion and RNA extraction.

### 2.10. Total RNA Extraction from Msm MLP, 0.8 OD, and 1 h Ap_6_A-Treated Cultures

Total RNA from the *Msm* cells harvested at 0.6 OD (MLP), 0.8 OD, and 1 h Ap_6_A-treated cultures were extracted using hot phenol method [42]. The cell pellets were crushed homogenously, using micro-pestle without thawing, using liquid nitrogen, and lysed in the lysis buffer containing 100 mM sodium acetate (Sigma-Aldrich, Saint Louis, MO, USA), pH 5.2, 10 mM EDTA (Sigma-Aldrich, Saint Louis, MO, USA), pH 8, 5 mM vanadyl ribonucleosides complex (VRC) (Sigma-Aldrich, Saint Louis, MO, USA), and 1% sodium dodecyl sulfate (SDS) (Sigma-Aldrich, Saint Louis, MO, USA), in RNase-free double-distilled water. Equal volumes of crushed cell pellet in the lysis buffer and hot phenol (phenol; Sisco Research Laboratories Pvt. Ltd., Mumbai, India) (phenol saturated with 100 mM sodium acetate, pH 5.2) were mixed gently and kept at 65 °C for 10 min, with intermittent mixing following centrifugation at ~12,000× *g*, for 10 min at 4 °C. The aqueous layer was collected and transferred to 1:1 ratio of ice-cold phenol (pH 5.2): chloroform (EMSURE® Merck, Darmstadt, Germany), extracted by mixing well, and centrifuged at ~12,000× *g* for 10 min at 4 °C. The aqueous phase was extracted with equal volume of ice-cold chloroform twice and the aqueous phase was collected by centrifugation at ~12,000× *g* for 10 min at 4 °C. The aqueous phase was mixed well with one-tenth the volume of 3 M sodium acetate, pH 5.2, and 2.5 volumes of 95% ice-cold ethanol, and the total RNA was precipitated overnight at −75 °C. The samples were centrifuged at ~12,000× *g* for 20 min at 4 °C. The pellet was washed twice with 70% ethanol containing 10 mM sodium acetate (pH 5.2) and centrifuged at ~12,000× *g* for 10 min at 4 °C and kept for air drying. The air-dried pellet was dissolved in DEPC-treated, RNase-free water and the total RNA was quantitated using nano spectrophotometer (Thermo Scientific NanoDrop 2000 spectrophotometer, Wilmington, DE, USA), using DEPC treated double distilled water as the blank. DNase I (50 U/µL; Thermo Fisher Scientific, Carlsbad, CA, USA) was used to remove DNA contamination in the RNA sample, using manufacturer’s protocol. The absence of DNA was confirmed using PCR for 16S rRNA gene (Supplementary Table S2).

### 2.11. cDNA Preparation and Real Time PCR

cDNAs specific for *MSMEG_2932, MSMEG_2933, MSMEG_2934, MSMEG_2935*, and *MSMEG_2936* genes were prepared using 100 ng of RNA and 500 nM of gene-specific reverse primer (Supplementary Table S2) in the presence of 0.5 mM dNTP mix (Thermo Scientific, Vilnius, Luthuania), 0.8 U of RevertAid premium reverse transcriptase (Thermo Scientific, Carlsbad, CA, USA) and 0.8 U of RiboLock (Thermo Scientific, Vilnius, Luthuania) in the presence of 1× RT buffer in RNase-free water. The reactions were carried out in SureCycler 8000 (PCR machine from Agilent Technologies, Penang, Malaysia) with denaturation at 65 °C for 5 min, annealing at 56 °C for 30 min, and inactivation of enzyme at 85 °C for 10 min. The cDNA thus obtained was used for quantitative PCR (qPCR) in CFX96 real-time PCR detection System (Bio-Rad, Hercules, CA, USA) using 10 µL of EvaGreen qPCR Mastermix-ROX (G-Biosciences^®^, Saint Louis, MO, USA), 500 nM each of forward and reverse primer, and 2 µL of cDNA per well per reaction, in a 96-well PCR plate. The values were analyzed using Bio-Rad CFX manager 3.1 (Bio-Rad, Foster City, CA, USA). The values obtained for each gene were normalized against 16S rRNA and the corresponding gene in the MLP sample [43]. For every gene analyzed, technical triplicates were made, and the experiment was performed in biological duplicates. Reaction conditions for the qPCR involved initial denaturation at 95 °C for 5 min, followed by amplification with 40 cycles of denaturation at 95 °C for 10 s, annealing at 56 °C for 20 s, and extension at 72 °C for 20 s. The comparative Ct (∆∆Ct) method was used for the calculation of fold-change in the expression levels of mRNA [44].

### 2.12. Generating Msm Knockout Strains of MSMEG_2932, MSMEG_2933, and MSMEG_2936

The construction of recombinant plasmids containing Allelic Exchange Substrates (AESs) were carried out using primers containing restriction endonuclease sites for directional cloning of the homologous flanking regions (here onwards ‘homologous flanking regions’ will be referred to as ‘flank’) in pYUB854 plasmid (Supplementary Table S1) [45,46]. The entire workflow of the generation of the knockout mutants of the three genes, *MSMEG_2932*, *MSMEG_2933*, and *MSMEG_2936* has been given in the Schemes S2–S7. The AESs were prepared using the protocol [47], with slight modifications. The standard cloning and sequence conformation procedures were performed using pBS-KS vector, as described [48,49]. The pYUB854-Msm-MSMEG-2932-KO was constructed using flank-1 containing 560 bp upstream and flank-2 containing 500 bp downstream of *MSMEG_2932* gene, which were PCR amplified using forward and reverse primers for flank-1 and flank-2 (Supplementary Table S3). The flank-1 and flank-2 of *MSMEG_2932* AES were ligated to pYUB854 using sequential ligation reactions as follows. The pYUB854 and flank-1 were digested with KpnI (Thermo Scientific, Vilnius, Luthuania) and XbaI (Thermo Scientific, Vilnius, Luthuania) and ligated. To obtain pYUB854-MSMEG-2932_flank-1, the ligated product was taken to transform *E. coli* JC10289 cells [30] (Supplementary Table S1). Now, this plasmid containing flank-1 construct and PCR amplified flank-2 were digested with XhoI (Thermo Scientific, Vilnius, Luthuania) and SpeI (Thermo Scientific, Vilnius, Luthuania), and ligated. *E. coli* JC10289 was then transformed with the construct containing flank-1 and flank-2 to obtain pYUB854-MSMEG-2932-KO plasmid DNA (Supplementary Table S1).

Similarly, the pYUB854-Msm-MSMEG-2933-KO was constructed using flank-1 containing 618 bp upstream and flank-2 containing 519 bp downstream of *MSMEG_2933* gene, which were PCR amplified using forward and reverse primers for flank-1 and flank-2 for *MSMEG_2933* gene (Supplementary Table S3). The flank-1 and flank-2 of *MSMEG_2933* AES were ligated to pYUB854 using sequential ligation reactions as follows. The pYUB854 and flank-1 were digested with KpnI and XbaI and ligated. *E. coli* JC10289 cells were transformed with the ligated product to obtain pYUB854-MSMEG-2933_flank-1 plasmid. Now this plasmid containing flank-1 construct and PCR amplified flank-2 were digested with NheI (Thermo Scientific, Vilnius, Luthuania) and NcoI (Thermo Scientific, Vilnius, Luthuania), and ligated. *E. coli* JC10289 cells were transformed with the construct containing flank-1 and flank-2 to obtain pYUB854-MSMEG-2933-KO plasmid DNA (Supplementary Table S1).

Similarly, the pYUB854-Msm-MSMEG-2936-KO was constructed using flank-1 containing 600 bp upstream of *MSMEG_2936* gene and flank-2 containing 500 bp downstream of *MSMEG_2936* gene, amplified using primers MSMEG2936-KO-flank1-f and MSMEG2936-KO-flank-1-r, for flank 1, and MSMEG2936-KO-flank2-f and MSMEG2936-KO-flank2-r for flank-2, respectively (Supplementary Table S3). pYUB854 and flank-1 were first digested with KpnI and XbaI, and ligated. *E. coli* JC10289 was transformed with the ligation reaction to obtain pYUB854-MSMEG-2936-flank1. The pYUB854-MSMEG-2936-flank1 plasmid DNA and PCR amplified flank-2 were digested with NcoI and HindIII (Thermo Scientific, Vilnius, Luthuania), and ligated. Ultracompetent *E. coli* JC10289 cells were transformed with ligation reaction mixture to obtain pYUB854-MSMEG_2936-KO plasmid DNA (Supplementary Table S1).

The *Msm* electrocompetent cells were prepared as described [50]. In brief, *Msm* cells at 0.8 OD were incubated on ice for 90 min and the cell pellet was collected by centrifugation at ~2000× *g*, 4 °C, for 10 min. The cell pellet was resuspended in autoclaved double-distilled water with 10% glycerol in half the original volume of the culture. The cell pellet was resuspended gently using Pipetman with 1/4th, 1/8th, and finally 1/20th volume of 10% glycerol in autoclaved double-distilled water, with centrifugation at ~2000× *g*, 4 °C, for 10 min, at each step. Aliquots of 100 µL each were prepared and stored at −75 °C.

The *Msm* electrocompetent cells were electroporated with pJV53 plasmid DNA (Supplementary Table S1), at 2500 V, 1000 Ω and 25 µF electrical pulse (Bio-Rad Gene Pulser Xcell, Bio-Rad, Hercules, CA, USA) in the 2 mm pre-chilled cuvette (Bio-Rad, Hercules, CA, USA). The cells were recovered and plated on Middlebrook 7H10 agar containing 0.2% glycerol and kanamycin (20 µg mL^−1^) (Sigma-Aldrich, Saint Louis, MO, USA). The construct thus obtained was *Msm*:pJV53 recombineering strain and was cultured in Middlebrook 7H9 broth containing 0.2% glycerol, 0.05% tween 80, 0.2% succinic acid (Sigma-Aldrich, Saint Louis, MO, USA) and kanamycin (20 µg mL^−1^), and incubated at 37 °C, 170 rpm for 48 h. The saturated culture was inoculated into Middlebrook 7H9 broth containing 0.2% glycerol, 0.05% Tween 80 0.2% succinic acid and kanamycin (20 µg mL^−1^), and incubated at 37 °C, 170 rpm until 0.4 OD. Then, 0.2% acetamide was added at 0.4 OD and kept at 37 °C, 170 rpm for 3 h [45]. After 3 h, the recombineering strain *Msm*:pJV53 was used for the preparation of electrocompetent cells, as described [50].

The pYUB854-Msm-MSMEG_2932-KO, pYUB854-Msm-MSMEG_2933-KO, and pYUB854-Msm-MSMEG_2936-KO plasmids (Supplementary Table S1), were digested with KpnI and SpeI (for pYUB854-Msm-MSMEG_2932-KO), KpnI and NcoI (for pYUB854-Msm-MSMEG_2933-KO), and KpnI and HindIII (for pYUB854-Msm-MSMEG_2936-KO), and the respective AES was gel purified (GeneJET gel extraction kit, Thermo Scientific, Vilnius, Luthuania). One hundred ng of linear AESs containing flank−1 and flank-2, flanking *hyg^R^* cassette and *res* sites, were used to electroporate *Msm*:pJV53 electrocompetent cells. The transformants were recovered with Middlebrook 7H9 medium containing 0.2% glycerol, 0.05% Tween 80, and 1x ADS (0.5% BSA, 0.75% dextrose, and 0.08% NaCl), at 37 °C for 6 h with shaking at 170 rpm and plated on Middlebrook 7H10 agar plate containing hygromycin (150 µg mL^−1^) (Sigma-Aldrich, Saint Louis, MO, USA). The genomic DNA was extracted from the colonies and were confirmed using PCR with specific primers (Supplementary Table S3) for the replacement of the respective gene with *hyg^R^* cassette.

These knockout strains, *Msm MSMEG_2932* KO, *Msm MSMEG_2933* KO, and *Msm MSMEG_2936* KO, were used to construct growth curve and determine SCD/ACD proportion with change in glycerol concentration at different growth phases using Middlebrook 7H9 medium containing 0.2% glycerol and 0.05% Tween 80, as the culture medium and incubating at 37 °C, 170 rpm. One ml each of the respective secondary culture was harvested at every 0.2 OD, at different growth phases from 0.2 OD at every 0.2 OD interval, until stationary phase. Glycerol concentration in the culture medium and ACD:SCD proportions were measured as described earlier.

### 2.13. Generating Genome Integrated Wild Type Gene Complemented Strains of the Msm Knockout Mutants MSMEG_2932 KO, MSMEG_2933 KO, and MSMEG_2936 KO

Genome integrating vector, pMV306 (Supplementary Table S1), was used for the complementation of knockout (KO) strains with the respective wild type gene under its own promoter, in the *MSMEG_2932* KO, *MSMEG_2933* KO, and *MSMEG_2936* KO strains. *MSMEG_2932*, *MSMEG_2933*, and *MSMEG_2936* were amplified from the WT *Msm* genomic DNA using the respective primers containing restriction enzyme sites (Supplementary Table S5). The PCR amplification was performed using Phusion high fidelity DNA polymerase (Thermo Fisher Scientific, Carlsbad, CA, USA). The PCR product of *MSMEG_2932* (881 bp), *MSMEG_2936* gene (1322 bp), and plasmid pMV306, were sequentially digested using XbaI and HindIII. Similarly, the PCR product of *MSMEG_2933* gene (974 bp) and plasmid pMV306 were sequentially digested using XbaI and EcoRI (New England Biolabs, Ipswich, MA, USA). The ligated products were used to transform calcium chloride competent *E. coli* JM109 cells (Supplementary Table S1). The recombinant *E. coli* JM109 cells were selected on LB agar containing 30 µg/mL kanamycin. The recombinant plasmids—pMV306-*MSMEG_2932*, pMV306-*MSMEG_2933*, and pMV306-*MSMEG_2936* (Supplementary Table S1), were isolated from the respective transformant *E. coli* JM109 cells, using GeneJET Plasmid Miniprep Kit (Thermo Fisher Scientific, Carlsbad, CA, USA) and used for electro-transformation of electrocompetent respective KO strains devoid of recombineering plasmid pJV53 (Supplementary Table S1). The *Msm* KO strains without recombineering plasmid pJV53 were obtained by growing the KO strains in Middlebrook 7H9 media containing 0.2% glycerol and 0.05% Tween 80, at 37 °C, at 170 rpm for 2 days up to saturation. Following this, an aliquot of the saturated culture was sub-cultured at a 1:10,000 ratio in fresh Middlebrook 7H9 medium and grown up to saturation for 2 days [44]. Aliquots of such culture were plated on 150 µg/mL hygromycin containing Middlebrook 7H11 agar (BD, Sparks, MD, USA) with 0.2% glycerol and 10% sucrose to cure the plasmid pJV53 [51]. The 7H11 agar plates were incubated at 37 °C, until colonies appear. The colonies thus obtained were patch-plated on to 7H11 agar containing 150 µg/mL hygromycin and 30 µg/mL kanamycin plates, respectively. The colonies appeared on hygromycin but absent on kanamycin containing plate, were used for preparing electrocompetent cells devoid of pJV53. The electrocompetent cells prepared [50], for *Msm MSMEG_2932* KO w/o pJV53, *MSMEG_2933* KO w/o pJV53, and *MSMEG_2936* KO w/o pJV53, were electroporated with pMV306-*MSMEG_2932*, pMV306-*MSMEG_2933*, and pMV306-*MSMEG_2936* plasmids, respectively (Supplementary Table S1). All three electrocompetent cells were electroporated individually with pMV306 plasmid (without any integrant) also, which was used as the respective vector control strain for respective complemented strains (Supplementary Table S1). The electroporation was performed at 2500 V, 1000 Ω, and 25 µF electrical pulse in the 2 mm pre-chilled cuvette. The cells were recovered with 2 mL 7H9 media containing 10% ADS supplement (0.5% BSA, 0.75% dextrose, and 0.08% NaCl) at 37 °C for 6 h. The cells were plated on Middlebrook 7H11 agar containing 30 µg/mL kanamycin and incubated at 37 °C, until colonies appeared. The colonies thus obtained, for each KO strain contained genome integrated respective gene, were used as the experimental system (Supplementary Table S1) to construct growth curve and determine SCD/ACD proportions with change in glycerol concentration at different growth phases, as described earlier.

## 3. Results

### 3.1. Experimental Rationale and Strategy

The change in the glycerol concentration from 0.2% original concentration, which is the main carbon source in Middlebrook 7H9 growth medium [28], was first determined until it got depleted vis-à-vis the progression of the growth of the culture. The presence of whole bacterial population may be critical for the cells to respond to the levels of glycerol as the decision for the change in ACD:SCD proportions might be taken at the population level. In fact, we had earlier shown that Ap_6_A, which is synthesized and secreted into the medium by *Msm* and *M. tuberculosis* cells, acts on the cells to induce ACD [37]. This possibility has been attended to in our entire study as we used flask-grown cultures with the medium containing only glycerol as the major carbon source in Middlebrook 7H9 medium [28]. This would not have been possible in microfluidics channels where the individual cells may suffer stress due to isolation and lack of inter-cellular communication. This is relevant, as fitness benefits in quorum sensing bacterial populations have been found to be high at higher bacterial density [52].

In parallel, at different concentrations of glycerol in the growth medium, the proportions of *Msm* cells dividing by ACD and SCD were determined by measuring lengths of the sister–daughter cells about to complete constriction/division. The ACD and SCD proportions were determined for the cells in the medium containing replenished glycerol also. Further, the ACD and SCD proportions were examined for the cells transferred from one OD of growth to the culture supernatant from another OD of growth, which differed in glycerol concentration. All of the measurements of the proportions of the cells dividing by ACD:SCD were made on the constriction-ending dividing mother cells after fixation on slides. These measurements of ACD:SCD proportions showed only negligible difference as compared to the ACD:SCD proportions measured using live cells [37]. Finally, to identify the probable genes involved in the changing proportions of the cells between the ACD and SCD, *Msm* knockout strains for three specific genes were generated and their ACD:SCD proportions under different glycerol concentrations were determined. Based on the observations, a model was proposed for the change of proportions of the *Msm* cells dividing by ACD/SCD in response to glycerol levels.

### 3.2. Growth of Msm Cells Vis-à-Vis Glycerol Concentration in the Medium

**Figure 1:**
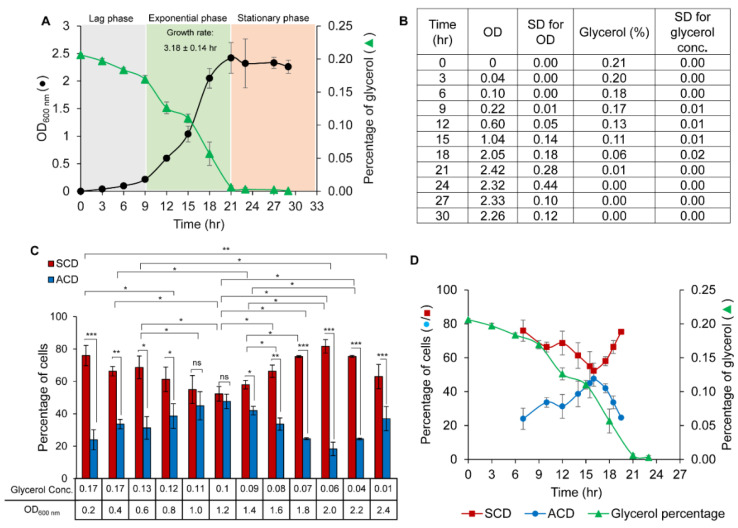
Growth curve of *Msm* cells and glycerol concentration in the medium. Percentage of the *Msm* cells undergoing ACD and SCD at different OD values and glycerol concentration. (**A**) (●) Growth curve of *Msm* cells grown in Middlebrook 7H9 liquid medium containing 0.2% glycerol and 0.05% Tween 80 and (▲) Glycerol concentration (percentage) in the culture medium with respect to OD and time; *n* = 3. (**B**) The levels of glycerol (with ± SD) at 3 h interval time points with the respective OD values of *Msm* growth from which the graph (**A**) was constructed (*n* = 3). (**C**) Bar graph represents the percentage of cells dividing by ACD (blue bar) and SCD (red bar) at different glycerol concentrations and OD values of cells grown in Middlebrook 7H9 liquid medium containing 0.2% glycerol and 0.05% Tween 80 (*n* = 300). (**D**) Comparison of the percentage of the cells undergoing (●) ACD and (■) SCD with respect to growth progression (in terms of time) dependent reduction in (▲) glycerol levels in the medium. The data shown are mean values ± s.d. of the biological triplicates. * *p* < 0.05, ** *p* < 0.01, *** *p* < 0.001, ns—no significance via two-tailed *t*-test.

The *Msm* cells grew with lag phase (mass doubling time of 2.46 ± 0.04 h), log phase, and stationary phase, and mass doubling time of 3.18 ± 0.14 h during exponential growth in Middlebrook 7H9 medium, containing 0.2% glycerol (specified concentration in the medium) and 0.05% Tween 80 (the concentration specified in the medium) (Figure 1A). The glycerol concentration in the mid-log phase primary culture used for inoculation was 0.33%, which was higher than 0.2% glycerol used in the medium as extra 0.2% glycerol was coming from the glycerol stock of *Msm* that contains 20% glycerol. Thus, a negligible quantity of 0.0033% of glycerol, from the 1% inoculum containing 0.33% added to 100 mL culture, will be present unavoidably in the secondary culture. The culture supernatant collected at different time points from three biological replicates of secondary culture showed steady decrease of glycerol levels with progress in growth phase and becoming almost nil at 21 h, coinciding with the onset of stationary phase (Figure 1A,B). The crossing of the glycerol level curve at 0.1% glycerol with the growth curve at around 1.2 OD of growth may be noted. The rate of depletion of glycerol in the medium in the initial OD_600 nm_ values up to 0.6 to 1.0 OD was at ~0.01% to 0.03% per 3 h (~one generation time), until it came to ~0.1% at 1.0 OD (Figure 1C, lower panel). Subsequently, the glycerol levels dropped steeply by 0.05% per 3 h from 1.0 OD_600 nm_ onwards until almost complete depletion (0.01%) at ~2.42 OD (Figure 1C, lower panel). These observations showed that glycerol utilization by mycobacteria changes with respect to growth phase, with a steep decline in the levels occurring from 0.1%. The calibration curve constructed for glycerol concentration, using known concentrations of glycerol showed a linear correlation between glycerol concentration and the absorbance, with the calculated R^2^ value of >0.99 (Supplementary Figure S1A,B).

### 3.3. Proportions of the Cells Dividing by ACD/SCD in Response to Glycerol Levels

Based on our earlier observations [9,10] and for a practically clear demarcation between ACD and SCD, all cell constrictions that generated daughter cells differing in length by ≥11% from the median were considered ACD. Similarly, all cell constrictions that generated daughter cells differing in length by <11% from the median were considered SCD. The relative error in the cell length measurements for three different non-dividing *Msm* cells, calculated from measurements performed multiple times (*n* = 20) from every experiment, was only 0.9 to 1.3% (Supplementary Figure S2). The primary culture at 0.6 OD, used for inoculation to generate secondary culture used for experiments showed ACD:SCD proportion of 20–30%:70–80%, as reported [10]. In the experiment assessing the proportions of the cells dividing by ACD/SCD vis-à-vis the OD, we started the measurements from 0.2 OD, as we did not want to measure less than 0.2 OD due to the possibility of less reliability. We checked the change in SCD/ACD percentage at every 0.2 increase in OD. Thus, there is no data for 3 h and 6 h for this experiment since the 0.2 OD came at 9 h post inoculation in the secondary culture (Figure 1C, bar graph). All of the growth phases from 0.2 OD to 2.4 OD in the secondary culture showed cells undergoing ACD and SCD. It was of interest to note that the ACD proportion steadily increased up to 1.0 OD, remained at the increased level up to 1.2 OD, and then steadily decreased up to 2.0 OD, with subsequent rise again after 2.0 OD (Figure 1C, bar graph). Reciprocally, the SCD proportion steadily decreased up to 1.0 OD, remained at the decreased level up to 1.2 OD and then steadily increased up to 2.0 OD, with subsequent decrease again (Figure 1C, bar graph).

### 3.4. Threshold Level of Glycerol for the Change of ACD/SCD Proportions

The change from ‘low-ACD/high-SCD’ to ‘equal-ACD:SCD’, and again to ‘low-ACD/high-SCD’ correlated with the decrease in glycerol levels with ~0.1% as the threshold concentration for the changeover. The ACD proportion steadily increased and the SCD proportion steadily decreased until the glycerol level reached ~0.1%, at which the ACD and SCD proportions were at ~50% each (Figure 1D). Subsequently, with the beginning of the further decrease in the glycerol level from ~0.1% in the medium (see Figure 1A,B), the ACD proportion began to decrease while the SCD proportion began to increase (Figure 1D). Thus, a switchover from high-ACD/low-SCD to low-ACD/ high-SCD in the population occurred when the glycerol level began to decrease further below ~0.1%. These observations revealed that the cell population seemed to be sensitive to 0.1% glycerol concentration in the medium and respond to further decrease in the concentration by altering the proportions of the cells dividing by ACD/SCD. Thus, ~0.1% glycerol seems to be the threshold concentration of glycerol for the switchover from high-ACD/low-SCD to low-ACD/high-SCD (at ~1.2 OD) (see Figure 1C, bar graph and Figure 1D).

### 3.5. Gaussian Distribution of Daughter-to-Mother Cell-Length Ratio for ACD/SCD

**Figure 2:**
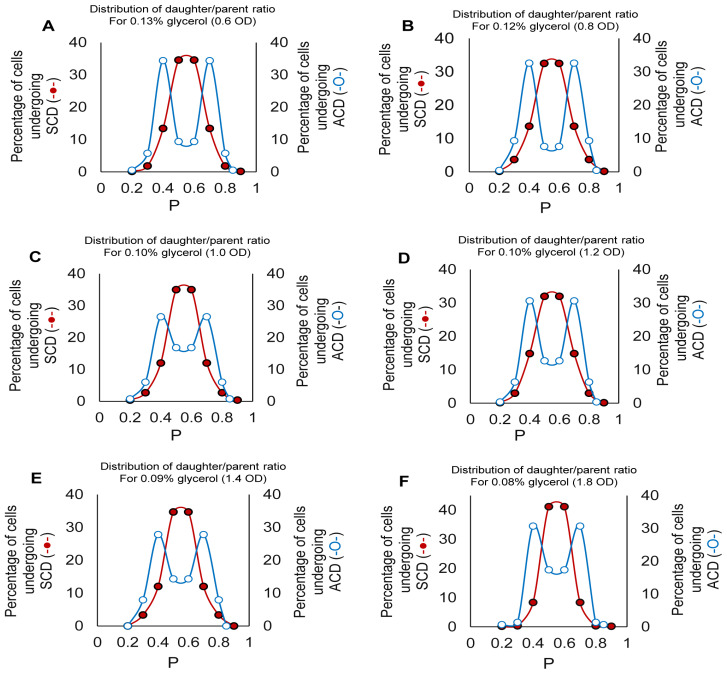
Gaussian distribution of daughter/parent ratio at different glycerol concentrations and OD values of growth. The daughter/parent ratio of distribution in (●) SCD and (o) ACD for (**A**) 0.6 OD (MLP), 0.13% glycerol; (**B**) 0.8 OD, 0.12% glycerol; (**C**) 1.0 OD, 0.11% glycerol; (**D**) 1.2 OD, 0.1% glycerol; (**E**) 1.4 OD, 0.09% glycerol; (**F**) 1.8 OD, 0.08% glycerol. The daughter/parent ratio of distribution in SCD shows unimodal and that in ACD shows bimodal distribution.

The distribution of daughter-to-mother cell-length ratio for the constricting cells (*n* = 300) from different OD values showed Gaussian distributions of a single peak around the *p*-value of 0.5 for SCD and of two peaks around the *p*-values of <0.5 and >0.5 for ACD (Figure 2A–F). The CV% for SCD was 4–6%, while that for ACD was 6–16% (Supplementary Table S4A,B). A high precision (CV < 10%) in the partition of one cell into two shows that the length variation among newly formed cells in the population is low, indicating SCD. Similarly, low precision (CV > 10%) in the partition of one cell into two shows that the length variation among newly formed cells is high, indicating ACD. The *w_n_* of the population of cells undergoing ACD showed higher value of >0.9, as compared to <0.9 for SCD, statistically indicating systematic asymmetric division (Supplementary Table S4C).

### 3.6. Maintenance of Glycerol at 0.1% Holds the ACD:SCD Proportion at ~50:50

**Figure 3:**
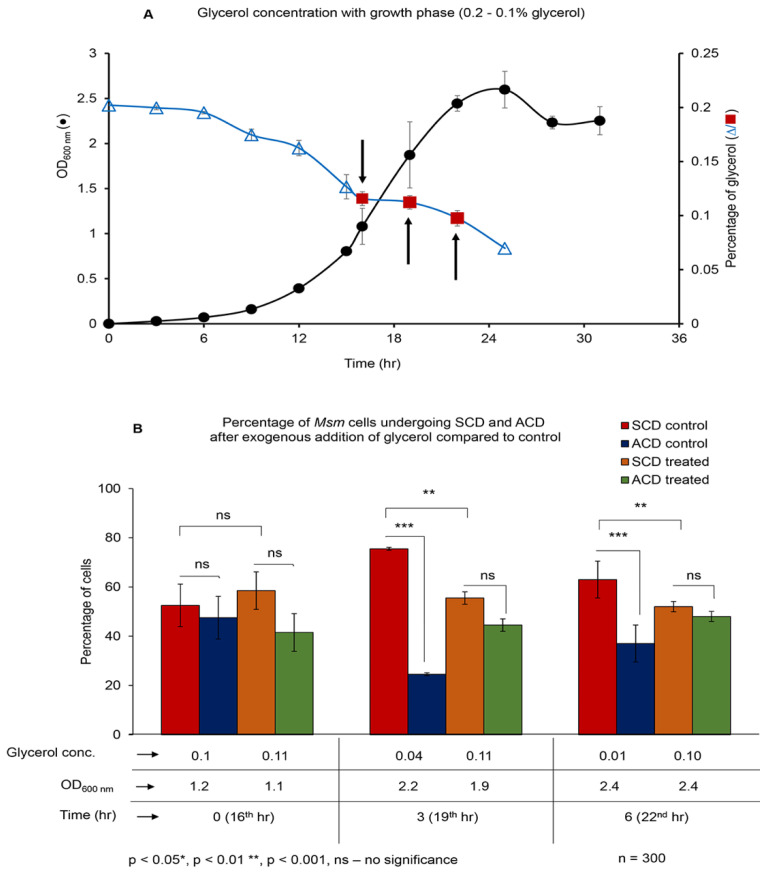
Proportion of the cells undergoing ACD/SCD after maintenance of glycerol at 0.1%, and growth curve with respect to OD. (**A**) (●) Growth curve of *Msm* cells cultured in Middlebrook 7H9 liquid medium containing 0.2% glycerol and 0.05% Tween 80, with respect to (∆) glycerol concentration and (■) after the exogenous addition of 0.05% glycerol at 16th, 19th, and 22nd h, as indicated by the arrows. (**B**) OD, glycerol concentration and proportion of SCD (red and orange bar) and ACD (blue and green bar) after the exogenous addition of glycerol at 0 h, 3rd h, and 6th h compared to the control culture without added glycerol at the same time point (*n* = 300. The data shown are for biological triplicates and represented with mean values ± s.d. *p* < 0.01 **, *p* < 0.001 ***, ns—no significance via two-tailed *t*-test).

It may be recalled that a change from the trend of ‘high-ACD/low-SCD’ to ‘low-ACD/high-SCD’ occurred when the glycerol levels began to decrease further from ~0.1%, where the ACD:SCD proportion was ~50:50 (see Figure 1C,D). Therefore, we wanted to find out whether the change could be prevented if the ACD:SCD proportion could be maintained at ~50:50 by retaining the glycerol levels at ~0.1%, through exogenous replenishment. The decrease in the glycerol levels, from 0.11% at 1.04 ± 0.14 OD at 15th h to further lower levels, was at the rate of 0.05% in every 3 h (see Figure 1B). Therefore, when the glycerol level reached ~0.1% in the culture supernatant at 15th h (at ~1.08 OD), 0.05% of autoclaved glycerol was added exogenously to the medium at 16th, 19th, and 22nd h, after determining the glycerol levels each time at 15th, 18th and 21st h (Figure 3A). The addition of 0.05% glycerol ensured that the glycerol equivalent to the exogenously added 0.05% will be utilized, thereby maintaining the glycerol levels at ~0.1% in the culture. This was evident from the levels of glycerol being maintained at ~0.11%, 0.11%, and 0.10% in the culture at the 15th h (0 h), 18th h (3rd h), and 21st h (9th h). This was reflected in the ACD:SCD proportions being maintained at ~50:50 at 1.1, 1.9, and 2.4 OD (compare Figure 3B). The respective control samples at the 16th h (0 h; 1.2 OD; 0.1% glycerol), 19th h (3rd h; 2.2 OD; 0.04% glycerol), and 22nd h (6th h; 2.4 OD; 0.01% glycerol), where the exogenous glycerol was not added, showed the ACD:SCD proportions as ~50:50 (at 0.1% glycerol), ~22:78 (at 0.04% glycerol), and ~38:62 (at 0.01% glycerol), respectively (Figure 3B; compare with Figure 1C). Thus, the maintenance of glycerol levels at ~0.1% through replenishment did not allow the change from high-ACD/low-SCD to low-ACD/high-SCD. Instead, the proportions were maintained at ~50:50. On the contrary, in the cultures where glycerol levels were not maintained at ~0.1%, the ACD:SCD proportions changed from ~50:50 to low-ACD:high-SCD again, as found in the first experiment (see Figure 1C,D). As in the case of the MLP cultures, the *Msm* cells grew with a lag phase, followed by a log phase, and later a stationary phase, with a mass doubling time of ~3.0 ± 0.04 h during exponential growth, in the glycerol-replenished culture (Figure 3A). The Middlebrook 7H9 media at 1X and 10X concentrations, added at the same quantity of exogenous glycerol (50 µL), showed the change in ACD/SCD proportion similar to that shown by the un-replenished culture (Supplementary Figure S3). It confirmed that the *Msm* cells do sense 0.1% glycerol as the threshold level in a sensitive manner vis-à-vis the ACD:SCD proportions. Further, it showed that the growth characteristics of the *Msm* cells were not affected by the maintenance of glycerol through exogenous addition during the experiment.

### 3.7. Starting the Culture with 0.1% Glycerol Concentration

**Figure 4:**
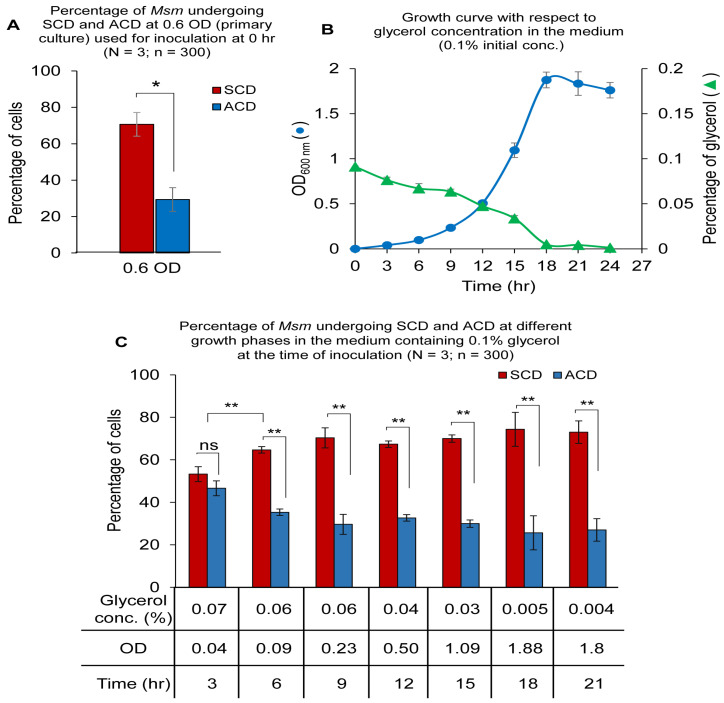
Growth curve of *Msm* cells, glycerol concentration in the medium, and proportion of the cells undergoing ACD/SCD in Middlebrook 7H9 liquid medium containing 0.1% glycerol and 0.05% Tween 80. (**A**) Percentage of the cells dividing by ACD/SCD at 0.6 OD of primary culture raised from glycerol stock and used for inoculation at 0 h. (**B**) (●) Growth curve of *Msm* with respect to (▲) glycerol concentration. (**C**) Percentage of the cells dividing by ACD/SCD at different glycerol concentrations and OD values at 3 h time intervals, *n* = 300. The data shown are for biological triplicates and represented with mean values ± s.d. ns—no significance, *p* < 0.05 *, *p* < 0.01 **, via two-tailed *t*-test.

Since 0.1% glycerol seemed to be a threshold concentration where the ACD:SCD proportion was maintained at ~50:50 and below 0.1% the trend switched over to ACD-decrease and SCD-increase, we wanted to find out the ACD and SCD proportions of *Msm* cells in the cultures that were started itself with 0.1% glycerol. One percent inoculum, from mid-log phase (0.6 OD) primary culture (Figure 4A), was inoculated into Middlebrook 7H9 medium containing 0.1% glycerol (instead of the formulated 0.2%) and 0.05% Tween 80. The *Msm* cells grew with lag phase, log phase and stationary phase, and generation time of ~3 h (Figure 4B). From 0 h to 3rd h post-inoculation, the ACD:SCD proportion was maintained at ~50:50 as expected in ~0.1% glycerol culture (see Figure 1C), although by then the glycerol levels had come down to 0.07% (Figure 4C). However, by the 6th h post-inoculation, with the glycerol level reaching ~0.06% at an OD of 0.09, the ACD:SCD proportion changed from 50:50 to ~38:62 (low-ACD, high-SCD) (Figure 4C), which was expected of a culture with 0.06% glycerol (see Figure 1C). Subsequently, with the steady decrease in glycerol levels to 0.04%, 0.03% and further below, the ACD:SCD proportion was maintained at ~38/30:62/70 (low-ACD/high-SCD), with gradual increase in OD (Figure 4B,C). These experiments confirmed that 0.1% glycerol is a threshold concentration where the bacilli maintain ACD:SCD at ~50:50, and that the bacilli would gradually change the equal ACD:SCD proportion to low-ACD/high-SCD when the glycerol levels decrease further below 0.1%. This experiment has once again confirmed that the bacilli are sensitive to 0.1% glycerol in the growth medium hinting that this threshold level of glycerol may have physiological significance.

### 3.8. The Influence of Glycerol in the Spent Medium on ACD/SCD Proportions

It was observed that the cultures approaching ~1.0–1.2 OD showed ACD-increase/SCD-decrease to reach ~50:50 at ~1.0–1.2 OD and subsequently showed ACD-decrease/SCD-increase from ~1.0–1.2 OD up to ~2 OD and then back to ACD-increase/SCD-decrease at still later OD values (see Figure 1C). Therefore, we wanted to find out whether the spent media from specific OD cultures influence the proportions of the cells dividing by ACD and SCD in other OD cultures. For this purpose, the cells from a specific OD culture with a specific ACD:SCD ratio were harvested and transferred to the supernatant from another OD culture containing partly spent glycerol. After the transfer, the OD of the culture, the glycerol levels, and the proportions of the cells dividing by ACD/SCD were determined at the 3rd, 6th, and 9th h post-transfer.

#### 3.8.1. The Response of 0.2 OD Cells in 1.2 OD Culture Supernatant

**Figure 5:**
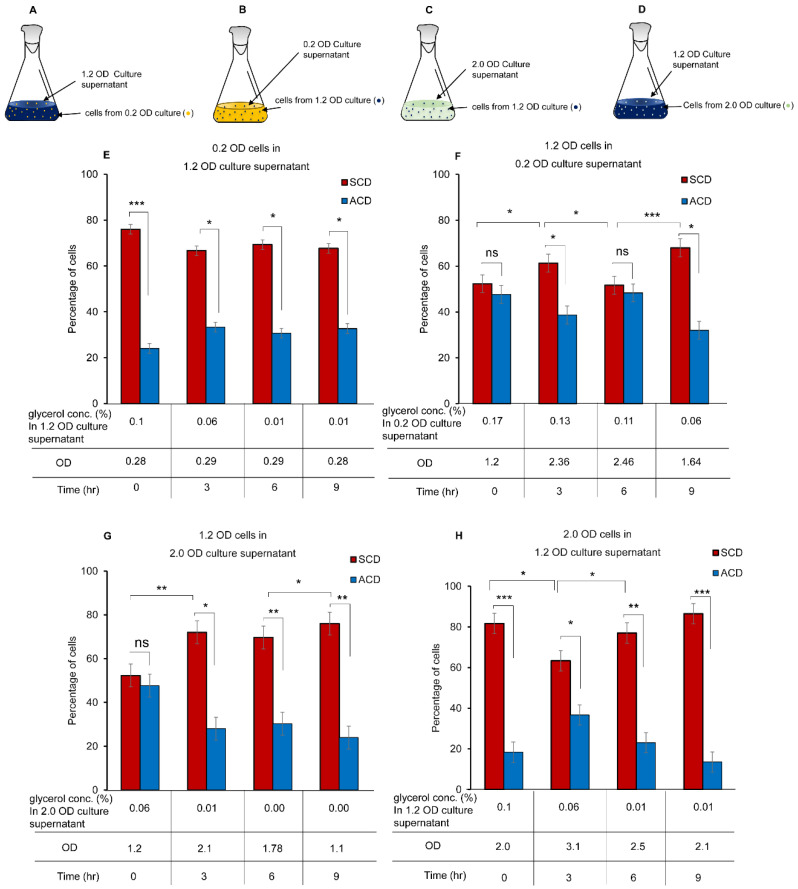
Proportions of the cells undergoing ACD/SCD after 3, 6, and 9 h when grown in the supernatant from another OD culture having different concentration of glycerol. (**A**–**D**) Cartoons for the transfer of the cells from one OD culture to the supernatant of another OD culture. Cartoons for the transfer of: (**A**) 0.2 OD cells to 1.2 OD culture supernatant; (**B**) 1.2 OD cells to 0.2 OD culture supernatant; (**C**) 1.2 OD cells to 2.0 OD culture supernatant; and (**D**) 2.0 OD cells to 1.2 OD culture supernatant. (**E**–**H**) The graphs for the ACD/SCD proportions, glycerol levels, and OD of the culture before and after transfer of the cells. (**E**) SCD and ACD proportion in 0.2 OD culture cells grown in 1.2 OD culture supernatant. (**F**) SCD and ACD proportion in 1.2 OD culture cells grown in 0.2 OD culture supernatant. (**G**) SCD and ACD proportion in 1.2 OD culture cells grown in 2.0 OD culture supernatant. (**H**) SCD and ACD proportion in 2.0 OD culture cells grown in 1.2 OD culture supernatant. (*n* = 300, the data shown are for the biological triplicates and represented with mean values ± s.d. ns—no significance, *p* < 0.05 *, *p* < 0.01 **, *p* < 0.001 *** via two-tailed *t*-test).

The cells from 0.2 OD culture with ~24:76 ACD:SCD proportion (see Figure 1C) were transferred to 1.2 OD culture supernatant (Figure 5A), where the ACD:SCD proportion was at ~50:50 in ~0.1% glycerol. Exposure of the cells for 3, 6, and 9 h showed that there was no significant change in the ACD and SCD proportions from that at 0.2 OD (at ~24:76 of ACD:SCD; see Figure 1C). The SCD proportion was retained high like that at the 0.2 OD control cells (Figure 5E, compare with Figure 1C). Lack of change in ACD/SCD proportion indicated lack of cell division as evident from the ~0.2 OD value of the cells maintained without change (Figure 5E). These observations showed that cell division might have got halted due to lack of nutrient in the medium due to the steady decrease in the glycerol concentration from 0.1% to 0.06%, and then to 0.01%.

#### 3.8.2. The Response of 1.2 OD Cells in 0.2 OD Culture Supernatant

In the reciprocal experiment, 1.2 OD cells, with the ACD:SCD proportion at ~50:50 in ~0.1% glycerol, were transferred to 0.2 OD culture supernatant (Figure 5B), where the ACD:SCD proportion was at ~24:76 in ~0.17% glycerol (see Figure 1C). The ACD:SCD proportion changed from ~50:50 to ~40:60 at the 3rd h post-transfer (at 0.13% glycerol) (Figure 5F), as expected of the cells in culture containing 0.12% glycerol. Subsequently, at the 6th h post-transfer when the glycerol concentration reached 0.11%, the ACD:SCD proportion changed from ~40:60 to ~50:50 (Figure 5F), which is as expected of the cells in 0.11% glycerol. Further, at the 9th h post-transfer, when the glycerol concentration reached 0.06%, the ACD:SCD proportion changed from ~50:50 to ~30:70 (Figure 5F), as expected of the cells in 0.06% glycerol (see Figure 1C). Over the 3rd and 6th h post-transfer, the OD values of the cells also increased (Figure 5F), as expected of the growth of the 1.2 OD cells in the nutrient rich 0.2 OD culture supernatant. Here it may be noted that even at high OD of 2.46, the ACD:SCD proportion was at ~50:50 indicating that the population is dividing by high ACD despite high OD of the culture. Thus, the ACD:SCD proportion was sensitive to the glycerol levels, but not influenced by the density of the culture.

#### 3.8.3. The Response of 1.2 OD Cells in 2.0 OD Culture Supernatant

In another experiment, the cells from 1.2 OD culture were transferred to 2.0 OD culture supernatant (Figure 5C). The 1.2 OD culture had glycerol levels at 0.1% with ~50:50 ACD:SCD ratio, while glycerol was 0.06% with ~18:82 ACD:SCD ratio in the 2.0 OD culture (see Figure 1C). By the 3rd h of exposure onwards, the ACD:SCD ratio changed over from ~50:50 to ~30:70 (Figure 5G), which is as expected of the cells in the 2.0 OD culture, (see Figure 1C). The ACD:SCD ratio of ~30:70 (high SCD) was maintained until the 9th h of exposure with the glycerol concentration reduced to almost nil (Figure 5G). An increase in the OD (from 1.2 to 2.1) could be observed by the 3rd h of exposure, probably due to cell elongation/division, which declined subsequently back to 1.1 OD by 9th h (Figure 5G).

#### 3.8.4. The Response of 2.0 OD Cells in 1.2 OD Culture Supernatant

In the reciprocal experiment, the cells from 2.0 OD culture, where glycerol was 0.06% with ACD:SCD ratio of ~18:82 (see Figure 1C), were transferred to the 1.2 OD culture supernatant (Figure 5D), where glycerol was 0.1% with ~50:50 ACD:SCD ratio. The ACD:SCD ratio of ~18:82 changed to ~40:60 by the 3rd h and subsequently showing a steady significant decrease to ~20:80 by the 6th h and then to ~15:85 by the 9th h of exposure at 2.1 OD (Figure 5H), as expected of 2.0 OD culture (see Figure 1C). The OD of the culture increased from 2.0 to 3.1 by the 3rd h of exposure and subsequently declined steadily to 2.1 OD by 9th h (Figure 5H). The cells seemed to have divided with higher proportion of SCD, with the higher cell number increasing the OD of the culture. These experiments indicated that mycobacterial cells, in response to the glycerol concentration in the culture medium, alter the proportions of the cells dividing by ACD/SCD.

### 3.9. Genes Influencing the ACD/SCD Proportions in Msm Cultures

**Figure 6:**
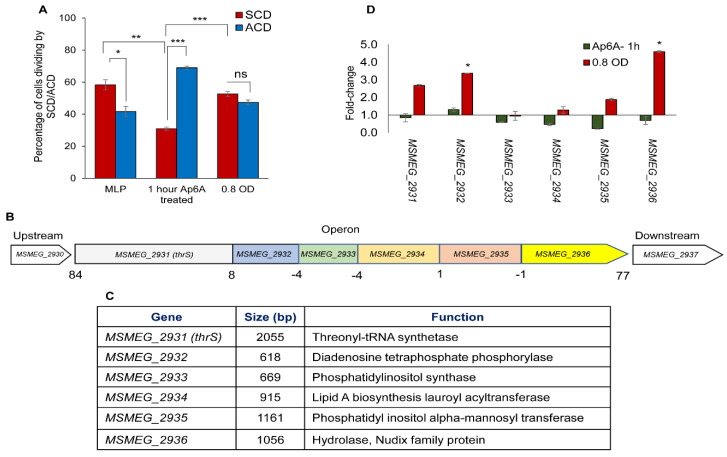
Proportion of the cells undergoing ACD:SCD in *M. smegmatis* MLP, Ap_6_A-exposed and 0.8 OD cultures, operon of six Ap_n_A/cell division related genes, and the fold-change in their expression. (**A**) Percentage of cells dividing by ACD/SCD in the cells from MLP, 166 pM Ap_6_A exposed cells for 1 h and 0.8 OD cultures (*n* = 300). The data shown are for biological triplicates, represented with mean values ± s.d. *p* < 0.05 *, *p* < 0.01 **, p < 0.001***, ns—no significance via two-tailed *t*-test). (**B**) *MSMEG_2931* (*thrS*)—*MSMEG_2936* operon. *MSMEG_2931* (*thrS*)—*MSMEG_2936* genes in the operon are shown in color boxes. The genes upstream and downstream of the operon are shown in white. The arrowhead indicates the transcription direction. The numbers appearing below the genes indicate the intergenic distance between two adjacent genes, where the negative number indicates the overlapping genes. The size of each boxes showing a gene in the operon are virtual representation of the gene size (not to scale) and color code for each box is random. (**C**) Genes, size (bp), and their function in *Msm*. (**D**) Fold-change in the expression of *MSMEG_2931*, *MSMEG_2932*, *MSMEG_2933*, *MSMSEG_2934*, *MSMSEG_2935*, and *MSMEG_2936* after 1 h exposure of MLP cells to Ap_6_A and from 0.8 OD cells. The real-time PCR data shown are from biological duplicate and represented with mean values ± s.d. Statistical analysis was performed using unpaired student’s *t*-test where asterisk (*) indicates *p*-value ≤ 0.05.

Having observed that proportions of *Msm* cells change between ACD and SCD modes of cell division in response to glycerol levels, we wanted to identify the genes that might influence the change in the proportions of the cells dividing by ACD/SCD vis-à-vis glycerol levels. In this regard, we had earlier reported that the biomolecule, diadenosine hexaphosphate (Ap_6_A), synthesized and secreted by *Msm* and *M. tuberculosis* into the growth medium, significantly increases the proportion of cells dividing by ACD in mycobacterial cultures [37]. In conformity with this finding, exposure of *Msm* MLP cells to 166 pM of synthetic Ap_6_A (Jena Bioscience, Jena, Germany) [37] for 1 h significantly changed the ACD/SCD proportion in the population, from ~40:60 at 0.6 OD (MLP) to 70:30, which was higher than the ACD:SCD proportion of ~50:50 at 0.8 OD (without Ap_6_A exposure) (Figure 6A), as reported [37]. Since Ap_6_A significantly changed the proportions of the cells dividing by ACD/SCD, it was quite likely that changes in the levels of Ap_6_A could change the ACD:SCD proportions. Therefore, the genes that are involved in the synthesis/degradation of Ap_6_A might in turn bring about changes in the proportions of *Msm* cells dividing by ACD/SCD.

In this regard, MSMEG_2932 in *M. smegmatis* [53] and its orthologue Rv2613c in *M. tuberculosis* [54] have been found to possess Ap_4_A phosphorylase activity, which converted Ap_4_A to ATP and ADP in the presence of phosphate. Meanwhile, purified lysyl-tRNA synthetase of *E. coli* was found to synthesize Ap_4_A and Ap_3_A in the presence of ATP, lysine and unfractionated tRNA [55]. Similarly, the synthesis of Ap_6_A from Ap4 (adenosine tetraphosphate) and ATP, in the presence of pyrophosphatase, was reported to be catalyzed by lysyl-tRNA synthetase (LysS) of *Myxococcus xanthus* [56]. Hinted by these findings and suspecting that these two genes might be functionally connected, we found that the Ap_4_A phosphorylase (*MSMEG_2932*) and threonyl-tRNA synthetase (*MSMEG_2931*) genes are part of an operon, which could be predicted as formulated [57]. This operon was found to consist of six genes, starting with *MSMEG_2931* as the first gene and *MSMEG_2936* encoding a Nudix family hydrolase as the last gene (Figure 6B,C). Further, since phosphoinositides, which include phosphatidyl inositol and its seven polyphosphate derivatives, are involved in cell division in eukaryotes [58], and mycobacteria being eubacteria, *MSMEG_2933* coding for phosphatidylinositol synthase and *MSMEG_2935* encoding phosphatidyl inositol alpha-mannosyl transferase also may have role in mycobacterial cell division. However, for want of information, we could not predict the involvement of lipid A biosynthesis lauroyl acyltransferase (*MSMEG_2934*), in any aspect of cell division.

Based on the possibility of a functional interlink of the genes in this operon, we hypothesized that the threonyl-tRNA synthetase (*MSMEG_2931*) might be converting Ap_4_A/Ap_4_ to Ap_6_A in the presence of ATP, like the lysyl-tRNA synthetase (LysS) of *Myxococcus xanthus* [56]. The *MSMEG_2936*, encoding a hydrolase of Nudix family of proteins, might be involved in determining Ap_4/6_A/Ap_4_ levels as Nudix family of proteins are involved in nucleotide hydrolysis, binding, and transfer [59,60,61,62]. These possibilities indicated that at least Ap_4_A phosphorylase (*MSMEG_2932*), phosphatidylinositol synthase (*MSMEG_2933*), and Nudix family hydrolase (*MSMEG_2936*) gene products might be involved in the synthesis/degradation of Ap_4_A/Ap_6_A. Hence, it was quite likely that disruption of these genes might affect the proportions of the cells dividing by ACD/SCD. In view of this hypothesis, the levels of expression of all six genes were determined using quantitative real time PCR.

The total RNA samples, extracted from 0.6 OD (MLP), 0.8 OD, and 1 h Ap_6_A exposed 0.6 OD (MLP) cells, were used to determine the levels of expression of all six genes using specific primers (Supplementary Table S2), keeping 0.6 OD (MLP) culture as the control sample. There were significantly high levels of *MSMEG_2932* and *MSMEG_2936* in the 0.8 OD sample but not in the Ap_6_A exposed samples (Figure 6D). The expression levels of all of the other genes were low and insignificant in the 0.8 OD sample. However, it was of interest to observe that Ap_6_A exposure reduced the expression levels of all six genes. The expression levels of *MSMEG_2933* (phosphatidylinositol synthase) at 0.8 OD was almost of same level as that of the control sample. The profile of higher expression these genes at 0.8 OD and reduced expression upon exposure to ACD-inducing Ap_6_A suggested that some of these genes, like Ap_6_A, might be involved in effecting the change of the proportions of the cells dividing by ACD/SCD. Based on these possibilities, knockout strains of *MSMEG_2932*, (diadenosine tetraphosphate phosphorylase), *MSMEG_2933*, (phosphatidylinositol synthase), and *MSMEG_2936* (Nudix family hydrolase) were generated, as discussed under Materials and Methods.

### 3.10. The Proportions of MSMEG_2932/2933/2936 KO Strains Dividing by ACD/SCD

**Figure 7:**
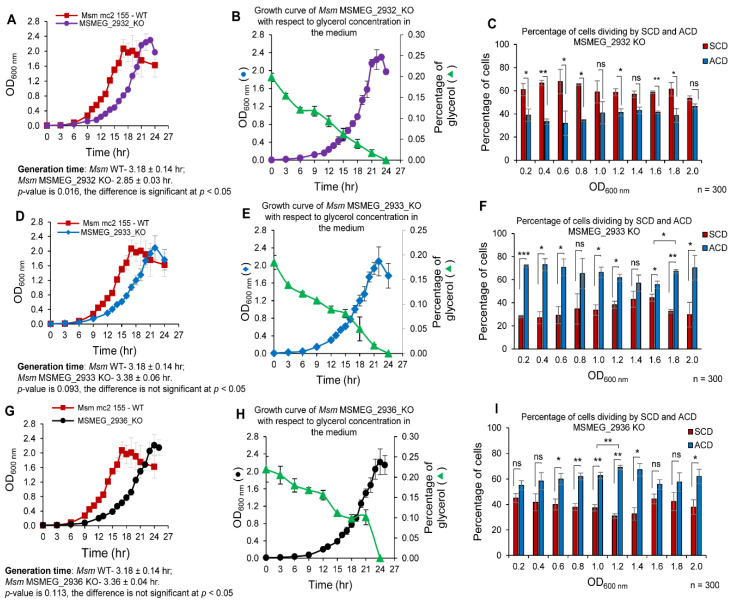
Growth profiles of *MSMEG_2932* KO, *MSMEG_2933* KO, and *MSMEG_2936* KO strains, in comparison to that of *Msm* WT, with respect to glycerol levels in the medium, and the proportion of the cells undergoing ACD/SCD. (**A**) Growth curve of *Msm* WT (■), and MSMEG_2932 KO (●). (**B**) Growth curve of MSMEG_2932 KO (●) with respect to the free glycerol levels in the medium (▲). (**C**) Bar graph for the percentage of cells dividing by SCD (red bar) and ACD (blue bar) of *Msm* MSMEG_2932 KO at different OD_600 nm_ values. (**D**) Growth curve of *Msm* WT (■) and MSMEG_2933 KO (◆). (**E**) Growth curve of MSMEG_2932 KO (◆) with respect to the free glycerol levels in the medium (▲). (**F**) Bar graph for the percentage of cells dividing by SCD (red bar) and ACD (blue bar) of *Msm* MSMEG_2933 KO at different OD_600 nm_ values. (**G**) Growth curve of *Msm* WT (■) and MSMEG_2936 KO (●). (**H**) Growth curve of MSMEG_2936 KO (●) with respect to the free glycerol levels in the medium (▲). (**I**) Bar graph for the percentage of cells dividing by SCD (red bar) and ACD (blue bar) of *Msm* MSMEG_2936 KO at different OD_600 nm_ values. The data shown are from biological triplicates and represented with mean values ± s.d. (*p* < 0.05 *, *p* < 0.01 **, *p* < 0.001 ***, ns—no significance via two-tailed *t*-test).

The respective growth curve of these strains in Middlebrook 7H9 medium, containing 0.2% glycerol and 0.05% Tween 80 was plotted using one ml culture harvested at different time intervals and was compared with the growth curve of WT *Msm* culture. All three KO strains grew with lag phase, log phase and stationary phase at a slower rate as compared to that of *Msm* WT (Figure 7A,D,G). The mass doubling time was found to be 2.85 ± 0.03 h for *Msm MSMEG_2932* KO, 3.38 ± 0.06 h for *Msm MSMEG_2933* KO, and 3.36 ± 0.04 h for *Msm MSMEG_2936* KO, as compared to the mass doubling time of 3.18 ± 0.14 h for the *Msm* WT strain determined in parallel. The culture supernatant collected at different time points from these three knockout strains showed steady decrease of glycerol levels with progress in growth and becoming almost nil at 24 h corresponding to the respective stationary phase (Figure 7B,E,H).

**Figure 8:**
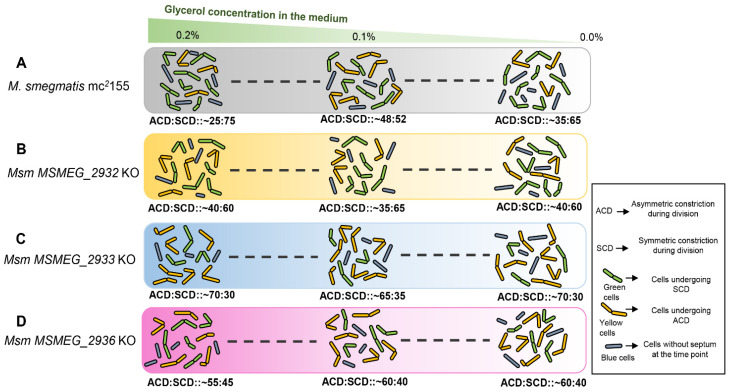
Model for the change in the ACD:SCD proportions in *M. smegmatis* cultures in response to glycerol levels in the growth medium. The ACD:SCD proportions are compared at 0.2% (initial), 0.1%, and almost nil at onset of stationary phase. The ACD:SCD proportions are shown for: (**A**) *M. smegmatis* mc^2^155, where the proportion changed from low-ACD/high-SCD to ~50:50 of ACD:SCD at 0.1% and again to low-ACD/high-SCD upon further decrease in glycerol levels; (**B**) *Msm MSMEG_2932* KO always showed low-ACD:high-SCD even at 0.1% glycerol concentration; (**C**) *Msm MSMEG_2933* KO; and (**D**) *Msm MSMEG_2936* KO strains always showed high-ACD:low-SCD proportion despite change in glycerol levels. Thus, *Msm*, with the loss of one of these genes, lost the ability to change ACD:SCD proportions in response to glycerol levels.

The SCD/ACD proportions were determined at every 0.2 OD difference. Despite the progression in growth phase and depletion of glycerol in the medium, *Msm MSMEG_2932* KO population always showed high SCD proportion at all of the time points (Figure 7C). On the contrary, the *Msm MSMEG_2933* KO population always showed high ACD proportion at all of the time points (Figure 7F). Like the *Msm MSMEG_2933* KO population, the *Msm MSMEG_2936* KO population also had showed high ACD at all of the time points, despite progression in growth phase, and change in glycerol levels in the medium (Figure 7I). These observations indicated that the knockout strains of *MSMEG_2932*, *MSMEG_2933*, and *MSMEG_2936* did not change their ACD:SCD proportions in response to glycerol levels. In other words, with the loss of either of these three genes, the cells seemed to have lost the ability to change ACD:SCD proportions in response to changes in glycerol levels, implying the involvement of these genes in the process. The genome integrated complemented strains of these three KO strains reversed the effect of the knockout and the change in ACD/SCD proportion was restored similar to that of *Msm* WT strain (Supplementary Figures S4–S6). The present study thus revealed the existence of a link among glycerol levels, sensing of the levels by a machinery involving at least the three genes studied, and response to the change in the levels of glycerol by changing the proportions of *Msm* cells dividing by ACD/SCD. These were graphically presented in a model (Figure 8).

## 4. Discussion

### 4.1. Link between Cell Division/Size and Nutrient Levels

There have been studies on the influence of nutrient status on cell division and/or cell-size that is tightly correlated with initiation of DNA replication. It was observed that the nutritional shift-up or change in nutrition causes a delay in cell division involving FtsZ [63]. A connection between cell division and nutrient levels was reported in *E. coli* and *Bacillus subtilis* where the genes OpgH in *E. coli* [64] and UgtP in *B. subtilis* [65,66] were involved in regulating cell-size vis-à-vis the onset of cell division events in response to nutrient levels. OpgH was found to localize to the nascent septal site, where it inhibited the assembly of the bacterial cell division protein FtsZ, thereby delayed cell division and increased cell size prior to DNA replication/segregation [67]. Similarly, in *B. subtilis*, UgtP localized to the nascent septal site in a nutrient-dependent manner and inhibited FtsZ assembly [65]. This process ensured that the cells reached the required mass and completed nucleoid segregation before the onset of cell division. The UgtP was found to be partly influenced by UDP-glucose as the intracellular proxy for nutrient availability, to control cell-size that is tightly linked to DNA replication/segregation for cell division.

### 4.2. Link between Cell Division and Diadenosine Polyphosphates (Ap_n_A)

Besides the above-described mechanisms related to nucleotide metabolism involving UDP-glucose at the very early stages of cell division, some earlier studies had demonstrated the existence of a link among diadenosine tetraphosphate (Ap_4_A), nutrient status, and cell division. Ap_4_A levels were found to be directly related to the levels of ATP/ADP ratio, which was sensitive to nutrient shift-down, but inversely related to the doubling time of mammalian cells [68]. In *E. coli*, Ap_4_A was found to control the timing of cell division [69]. A mutation in the *E. coli cfc* gene caused a reduction of the period between DNA replication and cell division in every cell cycle round, with a compensatory increase of the period between cell division and next round of nucleoid replication. This essentially uncoupled DNA replication and cell division and increased cell division frequency [69]. A mutation in the *apaH* (*cfcB1*) gene (Ap_4_A hydrolase) of *E. coli* [69], which is the orthologue of *MSMEG_2932*, was found to cause accumulation of Ap_4_A affecting motility and catabolite repression [70].

Taking these observations further, the present study showed the involvement of Ap_4_A phosphorylase gene (*MSMEG_2932*) in *Msm* (the orthologue of *E. coli apaH*) in determining the proportions of the cells dividing by ACD/SCD in response to glycerol levels in the growth medium. Like in the case of the *cfcB1* (*apaH*) mutant of *E. coli* [69], which increased cell division frequency, the deletion of Ap_4_A phosphorylase (*MSMEG_2932*) gene made the cells divide with significantly shorter mass doubling time (2.85 ± 0.03 h) as compared to that of the *Msm* WT strain (3.18 ± 0.14 h) (see Figure 7A). Further, the lack of change in the proportions of ACD:SCD upon the loss of this gene (*MSMEG_2932* KO strain), with SCD proportion remaining always high, further supported its involvement in the change of proportions of *Msm* cells in response to glycerol levels. Similar phenotype shown by the KO strains of phosphatidyl inositol synthase (*MSMEG_2933*) and Nudix family hydrolase (*MSMEG_2936*), but with ACD proportion remaining always high, also supported their role in deciding the ACD:SCD proportions of *Msm* cells in response to glycerol levels. Thus, these genes functionally linked the mode of cell division with glycerol levels.

### 4.3. The Change in ACD/SCD Proportions and 0.1% Glycerol

Mycobacterial populations have been found to show a range of cellular changes in response to different extents of nutrient depletion. Upon complete starvation of carbon and nitrogen sources, Mycobacterial cells have been found to lose acid-fast staining property [11], form ovoid shaped cells [13], and undergo sporulation in certain laboratory conditions [14,15,16]. Under extreme starvation and cryogenic stress conditions, *M. tuberculosis* and *M. bovis* cells were found to convert to L-forms, which were suggested to be an adaptation for survival and reproduction under highly unfavorable stress conditions [17,18,19]. Another instance of *M. smegmatis* cells sensing carbon source levels and bring alterations in the cell-length/size was reported where the *Msm* cells attained small cell morphotype while surviving in saline containing only traces of carbon sources [71]. On the contrary, under moderate conditions of nutrient limitation, such as in early stationary phase, reductive cell division was suggested to occur resulting in cell-size reduction [5,6].

The present study showed that 0.1% glycerol seemed to be a critical threshold value in the sensitivity of mycobacterial populations to the levels of glycerol in the medium for the modulation of the proportions of the cells dividing by ACD and SCD. The proportion of the cells dividing by ACD:SCD becoming ~50:50 showed that it was the changeover mark for the SCD/SCD proportions vis-à-vis glycerol levels. Before the glycerol levels became 0.1%, the SCD proportion would be high and decrease of the levels further from 0.1% made the bacteria to increase SCD proportion again and decrease ACD proportion. The sensitivity of mycobacteria to glycerol levels in the medium was further strengthened by the finding that the changes in the ACD:SCD proportion occurred in response to the glycerol levels in the culture supernatant despite the cells coming from a culture medium of a different glycerol level. These observations supported the possibility of a close link between ACD:SCD proportions and glycerol levels.

### 4.4. Why ACD:SCD Proportion Was Studied in Glycerol and Not in Other Possible Carbon Sources?

The 0.2% glycerol as the ideal carbon source for *M. tuberculosis* was originally formulated by Middlebrook in the 7H9 medium [28]. The change from low-ACD:high-SCD to equal ACD:SCD at 0.1% glycerol, and later again to low-ACD:high-SCD, with progressive depletion of glycerol in the Middlebrook 7H9 growth medium indicated that 0.2% concentration of glycerol was ideally suited for proper growth and division of mycobacteria. The 0.2% glycerol in the growth medium from the beginning ensured that double the concentration of the threshold level of 0.1% glycerol was available to begin with for maintaining low proportion of cells dividing by ACD. Maintenance of ACD at lower levels was important as the SCs, emerging from the ACD, were significantly more sensitive to antibiotics, oxidative, and nitrite stress than their sister NCs, although the SCs possessed significantly higher resister generation frequency [21,22,23]. Below and above 0.1% glycerol, the mode of cell division by low-ACD:high-SCD, which would yield low proportions of stress-susceptible SCs and high proportions of stress-tolerant NCs [21,22,23], would be an advantage for the population for survival under stress.

Glycerol, unlike glucose, has been found to be an ideal carbon source as it ensured the synthesis of higher content of lipids and polysaccharides that are characteristic of mycobacteria [72]. *M. tuberculosis* have been found to show abundant growth in glycerol which gets completely utilized without accumulated products or medium acidification [73,74,75]. The mass doubling time of the *Msm* cultures in our study was ~3 h in 0.2% glycerol. On the contrary, in the media containing pyruvate and acetate as the carbon source, *Msm* cells were found to have significantly slower mass doubling time of 8.6 and 4.8 h, respectively, as compared to that in glycerol (3.4 h) [76]. Further, the cells cultured in pyruvate and acetate showed less division asymmetry as compared to the cells grown in glycerol. The significantly slower mass doubling in the alternate carbon sources, such as pyruvate and acetate, indicated nutritional stress on the bacterial cells from the very beginning, which might get worse as growth and division progress. Hence, we did not examine the influence of pyruvate and acetate, or any other potential carbon sources for that matter, on ACD/SCD cell division pattern. Further, mycobacteria are known to have adapted to lipids (mostly triacylglycerol) as an evolutionarily important nutrient source enabling their lifestyle vis-à-vis living habitat [77]. Above all, the present study on the response of *Msm*, in terms of change in the proportions of the cells dividing by ACD:SCD and the genes involved therein, to yield higher proportion of more stress tolerant NCs and lower proportion of stress susceptible SCs, would enable further investigations on how mycobacteria would change their cell division strategy to maintain a high proportion of stress tolerant NCs in response to the nutrient status.

### 4.5. The Relevance of ACD:SCD Proportions’ Change for Survival under Stress

Observations made in many studies have shown a growing relevance for ACD in the response of mycobacteria to diverse types of stress such as antibiotics/oxidative/nitrite stress. For instance, the proportion of cells undergoing asymmetric division was found to increase from 20% in drug-susceptible strains to 42.2% and 44.4% in extensively drug-resistant TB (XDR-TB) and extremely drug-resistant TB (XXDR-TB) strains, respectively [78]. Short cells in the stationary phase were found to be more tolerant to oxidative stress and the tolerance was found induced during late exponential phase in a cell density dependent manner [5]. Short-sized cells (SCs) and the normal/long-sized cells (NCs), arising from ACD, were found to be inherently differentially susceptible, with the NCs being significantly more tolerant than the SCs, to oxidative and nitrite stress, and antibiotics [21,22,23]. At the same time, the SCs, despite being significantly more susceptible than the NCs to diverse stress conditions, were found to possess significantly high antibiotic resister generation frequency [22,23]. The NCs and the SCs, which are the products of ACD, have been found in the freshly diagnosed pulmonary tuberculosis patients’ sputum as well [9]. In view of these findings, the different proportions of the NCs and the SCs brought about by the alterations in the ACD:SCD proportions can effectively change the proportions of these subpopulations, thereby enabling the population to remain tolerant to or emerge as stress-resistant strains, under diverse stress conditions. Such a strategy dependent on carbon source status would be beneficial to the subpopulations in the tolerance/resistance/survival against antibiotics and other stress conditions, which in turn reveals the clinical significance of the present study.

## Data Availability

The data presented in this study are available in this article and Supplementary Materials only.

## Supplementary Materials

The following are available online at https://www.mdpi.com/article/10.3390/cells10051160/s1, Figure S1: Standard calibration curve for different glycerol concentrations. Figure S2: Percentage error in the measurement of three different *Msm* cells. Figure S3: Proportion of the cells undergoing ACD/SCD after addition of 1X Middlebrook 7H9 and 10X Middlebrook 7H9 every 3 h from 0.1% glycerol concentration. Figure S4: Growth profiles of *MSMEG_2932*_KO_pMV306-VC and *MSMEG_2932*_KO_pMV306-MSMEG_2932, with respect to glycerol levels in the medium, and the proportion of the cells undergoing ACD/SCD. Figure S5: Growth profiles of *MSMEG_2933_*KO_pMV306-VC and *MSMEG_2933*_KO_pMV306-MSMEG_2933, with respect to the glycerol levels in the medium, and the proportion of the cells undergoing ACD/SCD. Figure S6: Growth profiles of *MSMEG_2936_*KO_pMV306-VC and *MSMEG_2936*_KO_pMV306-MSMEG_2936, with respect to glycerol levels in the medium, and the proportion of the cells undergoing ACD/SCD. Table S1: Bacterial strains and plasmids with Supplementary references. Table S2: Oligonucleotide primers used for cDNA synthesis and real time PCR. Table S3: Oligonucleotide primers used for the gene replacement of *MSMEG_2932, MSMEG_2933*, and *MSMEG_2936* with res-*hyg*^R^-res and confirmation. Table S4: Coefficient of variation (CV), a measure of the dispersion in the location of the site of constriction from the mid-cell site. Table S5: Oligonucleotide primers used for the genome integrant complement generation of knockout strains of *MSMEG_2932* KO, *MSMEG_2933* KO, and *MSMEG_2936* KO. Scheme S1: *Msm* cell harvesting, fixation, DIC imaging, and data acquisition for measuring proportion of ACD and SCD. Scheme S2: *MSMEG_2932* gene knockout strategy. Scheme S3: *Msm MSMEG_2932*-KO clone confirmation using PCR and genomic DNA. Scheme S4: *MSMEG_2933* gene knockout strategy. Scheme S5: *Msm MSMEG_2933*-KO clone confirmation using PCR and genomic DNA. Scheme S6: *MSMEG_2936* gene knockout strategy. Scheme S7: *Msm MSMEG_2936-KO* clone confirmation using PCR and genomic DNA.

## Abbreviations

The following abbreviations were used in the manuscript.

ACD	asymmetric constriction during division
AES	allelic exchange substrate
CV	coefficient of variation
DDL	diacetyl dihydro lutidine
DEPC	diethyl pyrocarbonate
dNTP	deoxy nucleoside triphosphate
KO	knockout
MLP	mid-log phase
Msm	mycobacterium smegmatis
NCs	normal/long-sized cells
OD	optical density
PBS	phosphate buffered saline
PCR	polymerase chain reaction
PFA	paraformaldehyde
qPCR	quantitative polymerase chain reaction
RPM	revolutions per minute
SCD	symmetric constriction during division
SCs	short-sized cells
SDS	sodium dodecyl sulfate
VC	vector control
VRC	vanadyl ribonucleosides complex
XDR	extensively drug-resistant
XXDR	extremely drug-resistant

## References

[1] Ackermann M. (2015). A functional perspective on phenotypic heterogeneity in microorganisms. Nat. Rev. Microbiol..

[2] Hallez R., Bellefontaine A.F., Letesson J.J., De Bolle X. (2004). Morphological and functional asymmetry in alpha-proteobacteria. Trends Microbiol..

[3] Imaeda T. (1975). Ultrastructure of L-phase variants isolated from a culture of *Mycobacterium phlei*. J. Med. Microbiol..

[4] Khomenko A.G. (1987). The variability of *Mycobacterium tuberculosis* in patients with cavitary pulmonary tuberculosis in the course of chemotherapy. Tubercle.

[5] Smeulders M.J., Keer J., Speight R.A., Williams H.D. (1999). Adaptation of *Mycobacterium smegmatis* to stationary phase. J. Bacteriol..

[6] Thanky N.R., Young D.B., Robertson B.D. (2007). Unusual features of the cell cycle in mycobacteria: Polar-restricted growth and the snapping-model of cell division. Tuberculosis.

[7] Ryan G.J., Hoff D.R., Driver E.R., Voskuil M.I., Gonzalez-Juarrero M., Basaraba R.J., Crick D.C., Spencer J.S., Lenaerts A.J. (2010). Multiple *M. tuberculosis* phenotypes in mouse and guinea pig lung tissue revealed by a dual-staining approach. PLoS ONE.

[8] Zetola N.M., Modongo C., Moonan P.K., Ncube R., Matlhagela K., Sepako E., Collman R.G., Bisson G.P. (2014). Clinical outcomes among persons with pulmonary tuberculosis caused by *Mycobacterium tuberculosis* isolates with phenotypic heterogeneity in results of drug-susceptibility tests. J. Infect. Dis..

[9] Vijay S., Nagaraja M., Sebastian J., Ajitkumar P. (2014). Asymmetric cell division in *Mycobacterium tuberculosis* and its unique features. Arch. Microbiol..

[10] Vijay S., Mukkayyan N., Ajitkumar P. (2014). Highly deviated asymmetric division in very low proportion of mycobacterial mid-log phase cells. Open Microbiol. J..

[11] Nyka W. (1974). Studies on the effect of starvation on mycobacteria. Infect. Immun..

[12] Shleeva M.O., Mukamolova G.V., Young M., Williams H.D., Kaprelyants A.S. (2004). Formation of ‘non-culturable’ cells of *Mycobacterium smegmatis* in stationary phase in response to growth under suboptimal conditions and their Rpf-mediated resuscitation. Microbiology.

[13] Anuchin A.M., Mulyukin A.L., Suzina N.E., Duda V.I., El-Registan G.I., Kaprelyants A.S. (2009). Dormant forms of *Mycobacterium smegmatis* with distinct morphology. Microbiology.

[14] Ghosh J., Larsson P., Singh B., Pettersson B.M.F., Islam N.M., Sarkar S.N., Dasgupta S., Kirsebom L.A. (2009). Sporulation in mycobacteria. Proc. Natl. Acad. Sci. USA.

[15] Singh B., Ghosh J., Islam N.M., Dasgupta S., Kirsebom L.A. (2010). Growth, cell division and sporulation in mycobacteria. Antonie Leeuwenhoek.

[16] Lamont E.A., Bannantine J.P., Armien A., Ariyakumar D.S., Sreevatsan S. (2012). Identification and characterisation of a spore-like morphotype in chronically starved *Mycobacterium avium* Subsp. *Paratuberculosis* cultures. PLoS ONE.

[17] Slavchev G., Michailova L., Markova N. (2013). Stress-induced L-forms of *Mycobacterium bovis*: A challenge to survivability. New Microbiol..

[18] Markova N., Slavchev G., Michailova L. (2012). Unique biological properties of *Mycobacterium tuberculosis* L-form variants: Impact for survival under stress. Int. Microbiol..

[19] Markova N., Michailova L., Jourdanova M., Kussovski V., Valcheva V. (2008). Exhibition of persistent and drug tolerant L-form habit of *Mycobacterium tuberculosis* during infection in rats. Cent. Eur. J. Biol..

[20] Baek S.H., Li A.H., Sessetti C.M. (2011). Metabolic regulation of mycobacterial growth and antibiotic sensitivity. PLoS Biol..

[21] Vijay S., Nair R.R., Sharan D., Jakkala K., Mukkayyan N., Swaminath S., Pradhan A., Joshi N.V., Ajitkumar P. (2017). Mycobacterial cultures contain cell size and density specific sub-populations of cells with significant differential susceptibility to antibiotics, oxidative and nitrite stress. Front. Microbiol..

[22] Nair R.R., Sharan D., Sebastian J., Swaminath S., Ajitkumar P. (2019). Heterogeneity of ROS levels in antibiotic-exposed mycobacterial subpopulations confers differential susceptibility. Microbiology.

[23] Nair R.R., Sharan D., Ajitkumar P. (2019). A minor subpopulation of mycobacteria inherently produces high levels of reactive oxygen species that generate antibiotic resisters at high frequency from itself and enhance resister generation from its major kin subpopulation. Front. Microbiol..

[24] Dahl J.L. (2004). Electron microscopy analysis of *Mycobacterium tuberculosis* cell division. FEMS Microbiol. Lett..

[25] Aldridge B.B., Fernandez-Suarez M., Heller D., Ambravaneswaran V., Irimia D., Toner M., Fortune S.M. (2012). Asymmetry and aging of mycobacterial cells lead to variable growth and antibiotic susceptibility. Science.

[26] Joyce G., Williams K.J., Robb M., Noens E., Tizzano B., Shahrezaei V., Robertson B.D. (2012). Cell division site placement and asymmetric growth in mycobacteria. PLoS ONE.

[27] Middlebrook G., Cohn M.L. (1958). Bacteriology of tuberculosis: Laboratory methods. Am. J. Public Health.

[28] Snapper S.B., Melton R.E., Mustafa S., Kieser T., Jacobs W.R. (1990). Isolation and characterisation of efficient plasmid transformation mutants of *Mycobacterium smegmatis*. Mol. Microbiol..

[29] Yanisch-Perron C., Vieira J., Messing J. (1985). Improved M13 phage cloning vectors and host strains: Nucleotide sequences of the M13mp18 and pUC19 vectors. Gene.

[30] Csonka L.N., Clark A.J. (1979). Deletions generated by the transposon Tn10 in the *srl recA* region of the *Escherichia coli* K-12 chromosome. Genetics.

[31] Widdel F. (2007). Theory and measurement of bacterial growth. Di Dalam Grund. Mikrobiol..

[32] Hartman L. (1953). Rapid determination of glycerol by the potassium periodate method. J. Appl. Chem..

[33] Nash T. (1953). The colorimetric estimation of formaldehyde by means of the Hantzsch reaction. Biochem. J..

[34] White D.A., Miyada D.S., Nakamura R.M. (1974). Characterisation of the periodate oxidation of glycerol and related compounds, and application toward determination of serum triglyceride concentrations. Clin. Chem..

[35] Bondioli P., Bella L.D. (2005). An alternative spectrophotometric method for the determination of free glycerol in biodiesel. Eur. J. Lipid Sci. Technol..

[36] Kuhn J., Muller H., Salzig D., Czermak P. (2015). A rapid method for an offline glycerol determination during microbial fermentation. Electron. J. Biotechnol..

[37] Mukkayyan N., Sharan D., Ajitkumar P. (2018). A symmetric molecule produced by mycobacteria generates cell-length asymmetry during cell-division and thereby cell-length asymmetry during cell-division and thereby cell-length heterogeneity. ACS Chem. Biol..

[38] Powell E.O. (1964). A note on Koch & Schaechter’s hypothesis about growth and fission in bacteria. J. Gen. Microbiol..

[39] Marr A.G., Harvey R.J., Trentini W.C. (1966). Growth and division of *Escherichia coli*. J. Bacteriol..

[40] Treuba F.J. (1982). On the precision and accuracy achieved by *Escherichia coli* cells at fission about their middle. Arch. Microbiol..

[41] Geary R.C. (1935). The ratio of the mean deviation to the standard deviation as a test of normality. Biometrika.

[42] Wecker E. (1959). The extraction of infectious virus nucleic acid with hot phenol. Virology.

[43] Willems E., Leyns L., Vandesompele J. (2008). Standardisation of real-time PCR gene expression data from independent biological replicates. Anal. Biochem..

[44] Wang W., Chen K., Xu C. (2006). DNA quantification using EvaGreen and a real-time PCR instrument. Anal. Biochem..

[45] van Kessel J.C., Hatfull G.F. (2007). Recombineering in *Mycobacterium tuberculosis*. Nat. Meth..

[46] Bardarov S., Bardarov S., Pavelka M.S., Sambandamurthy V., Larsen M., Tufariello J., Chan J., Hatfull G., Jacobs W.R. (2002). Specialised transduction: An efficient method for generating marked and unmarked targeted gene disruptions in *Mycobacterium tuberculosis*, *M. bovis* BCG and *M. smegmatis*. Microbiology.

[47] Bibb L.A., Hatfull G.F. (2002). Integration and excision of the *Mycobacterium tuberculosis* prophage-like element, phiRv1. Mol. Microbiol..

[48] Alting-Mees M.A., Short J.M. (1989). pBluescript II: Gene mapping vectors. Nucleic Acids Res..

[49] Ausubel F., Kingston R. (1987). . Current Protocols in Molecular Biology.

[50] Goude R., Parish T. (2009). Electroporation of mycobacteria. Meth. Mol. Biol..

[51] Mao X.J., Yan M.Y., Zhu H., Guo X.P., Sun Y.C. (2016). Efficient and simple generation of multiple unmarked gene deletions in *Mycobacterium smegmatis*. Sci. Rep..

[52] Darch S.E., West S.A., Winzer K., Diggle S.P. (2012). Density-dependent fitness benefits in quorum-sensing bacterial populations. Proc. Natl. Acad. Sci. USA.

[53] Honda N., Kim H., Rimbara E., Kato A., Shibayama K., Mori S. (2015). Purification and functional characterisation of diadenosine 5′,5-P^1^,P^4^-tetraphosphate phosphorylases from *Mycobacterium smegmatis* and *Mycobacterium avium*. Protein Expr. Purif..

[54] Mori S., Shibayama K., Wachino J.I., Arakawa Y. (2010). Purification and molecular characterisation of a novel diadenosine 5′,5‴-P1,P4-tetraphosphate phosphorylase from *Mycobacterium tuberculosis* H37Rv. Protein Expr. Purif..

[55] Zamecnik P.C., Stephenson M.L., Janeway C.M., Randerath K. (1966). Enzymatic synthesis of diadenosine tetraphosphate and diadenosine triphosphate with a purified lysyl-sRNA synthetase. Biochem. Biophys. Res. Commun..

[56] Oka M., Takegawa K., Kimura Y. (2016). Lysyl-tRNA synthetase from *Myxococcus xanthus* catalyses the formation of diadenosine penta- and hexaphosphates from adenosine tetraphosphate. Arch. Biochem. Biophys..

[57] Dehal P.S., Joachimiak M.P., Price M.N., Bates J.T., Baumohl J.K., Chivian D., Fridland G.D., Huang K.H., Keller K., Novichkov P.S. (2010). Microbesonline: An integrated portal for comparative and functional genomics. Nucleic Acids Res..

[58] Cauvin C., Echard A. (2014). Phosphoinositides: Lipids with informative heads and mastermind functions in cell division. Biochim. Biophys. Acta.

[59] Brenner C., Pace H.C., Garrison P.N., Robinson A.K., Rosler A., Liu X.H., Blackburn G.M., Croce C.M., Huebner K., Barnes L.D. (1997). Purification and crystallisation of complexes modelling the active state of the fragile histidine triad protein. Protein Eng..

[60] Huebner K., Hadaczek P., Siprashvili Z., Druck T., Croce C.M. (1997). The *FHIT* gene, a multiple tumor suppressor gene encompassing the carcinogen sensitive chromosome fragile site, *FRA3B*. Biochim. Biophys. Acta.

[61] Lima C.D., Klein M.G., Hendrickson W.A. (1997). Structure-based analysis of catalysis and substrate definition in the HIT protein family. Science.

[62] Le Beau M.M., Drabkin H., Glover T.W., Gemmill R., Rassool F.V., McKeithan T.W., Smith D.I. (1998). An *FHIT* tumor suppressor gene?. Genes Chromosomes Cancer.

[63] Kepes F., D’Ari R. (1987). Involvement of FtsZ Protein in Shift-Up-Induced Division Delay in *Escherichia coli*. J. Bacteriol..

[64] Chien A.-C., Hill N.S., Levin P.A. (2012). Cell size control in bacteria. Curr. Biol..

[65] Weart R.B., Lee A.H., Chien A.-C., Haeusser D.P., Hill N.S., Levin P.A. (2007). A metabolic sensor governing cell size in bacteria. Cell.

[66] Chien A.-C., Zareh S.K.G., Wang Y.M., Levin P.A. (2012). Changes in the oligomerisation potential of the division inhibitor UgtP coordinates *Bacillus subtilis* cell size with nutrient availability. Mol. Microbiol..

[67] Hill N.S., Buske P.J., Shi Y., Levin P.A. (2013). A Moonlighting enzyme links *Escherichia coli* cell size with central metabolism. PLoS Genet..

[68] Rapaport E., Zamecnik P.C. (1976). Presence of diadenosine 5′,5″–P^1^,P^4^-tetraphosphate (Ap_4_A) in mammalian cells in levels varying widely with proliferative activity of the tissue: A possible positive “pleiotypic activator”. Proc. Natl. Acad. Sci. USA.

[69] Nishimura A., Moriya S., Ukai H., Nagai K., Wachi M., Yamada Y. (1997). Diadenosine 5′,5″–P^1^,P^4^-tetraphosphate (Ap_4_A) controls the timing of cell division in *Escherichia coli*. Genes Cells.

[70] Farr S.B., Arnosti D.N., Chamberlin M.J., Ames B.N. (1989). An *apaH* mutation causes AppppA to accumulate and affects motility and catabolite repression in *Escherichia coli*. Proc. Natl. Acad. Sci. USA.

[71] Wu M.-L., Gengenbacher M., Chung J.C.S., Chen S.L., Mollenkopf H.-J., Kaufmann S.H.E., Dick T. (2016). Developmental transcriptome of resting cell formation in *Mycobacterium smegmatis*. BMC Genom..

[72] Tepper B.S. (1968). Differences in the utilisation of glycerol and glucose by *Mycobacterium phlei*. J. Bacteriol..

[73] Dingle J.H., Weinzirl J. (1932). The biology of th tubercle bacillus. II. The asparagine and glycerol metabolism of the tubercle bacillus. J. Bacteriol..

[74] Wedum A.G. (1936). Glycerol and carbohydrate utilisation by *Mycobacterium tuberculosis*. J. Bacteriol..

[75] Hunter G.J.E. (1953). The oxidation of glycerol by mycobacteria. Biochem. J..

[76] Priestman M., Thomas P., Robertson B.D., Shahrezaei V. (2017). Mycobacteria modify their cell size control under sub-optimal carbon sources. Front. Cell Dev. Biol..

[77] Mali P.C., Meena L.S. (2018). Triacylglycerol: Nourishing molecule in endurance of *Mycobacterium tuberculosis*. J. Biosci..

[78] Farnia P., Masjedi M.R., Merza M.A., Tabarsi P., Zhavnerko G.K., Ibrahim T.A., Kuan H.O., Ghanavei J., Farnia P., Ranjbar R. (2010). Growth and cell-division in extensive (XDR) and extremely drug resistant (XXDR) tuberculosis strains: Transmission and atomic force observation. Int. J. Clin. Exp. Med..

