# Supplementary material for: Mycobacterial Populations Partly Change the Proportions of the Cells Undergoing Asymmetric/Symmetric Divisions in Response to Glycerol Levels in Growth Medium
Source: Cells. 2021 May 11;10(5):1160. doi: 10.3390/cells10051160 (PMC8151439; doi:10.3390/cells10051160)
Supplement: Supplementary file 1 [file cells-10-01160-s001.zip › cells-1062214-supplementary.pdf]

Supplementary Material

Mycobacterial populations partly change the proportions of the cells undergoing asymmetric/symmetric divisions in response to glycerol levels in the growth medium

Atul Pradhan, Nagaraja Mukkayyan, Kishor Jakkala, and Parthasarathi Ajitkumar

Department of Microbiology and Cell Biology, Indian Institute of Science, Bengaluru, Karnataka, India

Supplementary Materials: The Supplementary Materials contain **Figures S1-S6**, **Schemes S1-S7**, **Tables S1-S5**. **Figure S1**: Standard calibration curve for different glycerol concentrations. **Figure S2**: Percentage error in the measurement of three different *Msm* cells. **Figure S3**: Proportion of the cells undergoing ACD/SCD after addition of 1X Middlebrook 7H9 and 10X Middlebrook 7H9 every 3 hr from 0.1% glycerol concentration. **Figure S4**: Growth profiles of *MSMEG_2932*_KO_pMV306-VC and *MSMEG_2932*_KO_pMV306-MSMEG_2932, with respect to glycerol levels in the medium, and the proportion of the cells undergoing ACD/SCD. **Figure S5**: Growth profiles of *MSMEG_2933*_KO_pMV306-VC and *MSMEG_2933*_KO_pMV306-MSMEG_2933, with respect to the glycerol levels in the medium, and the proportion of the cells undergoing ACD/SCD. **Figure S6**: Growth profiles of *MSMEG_2936*_KO_pMV306-VC and *MSMEG_2936*_KO_pMV306-MSMEG_2936, with respect to glycerol levels in the medium, and the proportion of the cells undergoing ACD/SCD. **Scheme S1**: *Msm* cell harvesting, fixation, DIC imaging and data acquisition for measuring proportion of ACD and SCD. **Scheme S2**: Gene knockout

strategy. **Scheme S3**: *Msm MSMEG_2932*-KO clone confirmation using PCR and genomic DNA. **Scheme S4**: *MSMEG_2933* gene knockout strategy. **Scheme S5**: *Msm MSMEG_2933*-KO clone confirmation using PCR and genomic DNA. **Scheme S6**: *MSMEG_2936* gene knockout strategy. **Scheme S7**: *Msm MSMEG_2936-KO* clone confirmation using PCR and genomic DNA. **Table S1**: Bacterial strains and plasmids with Supplementary references. **Table S2**: Oligonucleotide primers used for cDNA synthesis and real time PCR. **Table S3**: Oligonucleotide primers used for the gene replacement of *MSMEG_2932, MSMEG_2933,* and *MSMEG_2936* with res-*hyg*^{R}-res and confirmation. **Table S4**: Coefficient of variation (CV), a measure of the dispersion in the location of the site of constriction from the mid-cell site. **Table S5**: Oligonucleotide primers used for the genome integrant complement generation of knock-out strains of *MSMEG_2932* KO, *MSMEG_2933* KO, and *MSMEG_2936* KO.

A

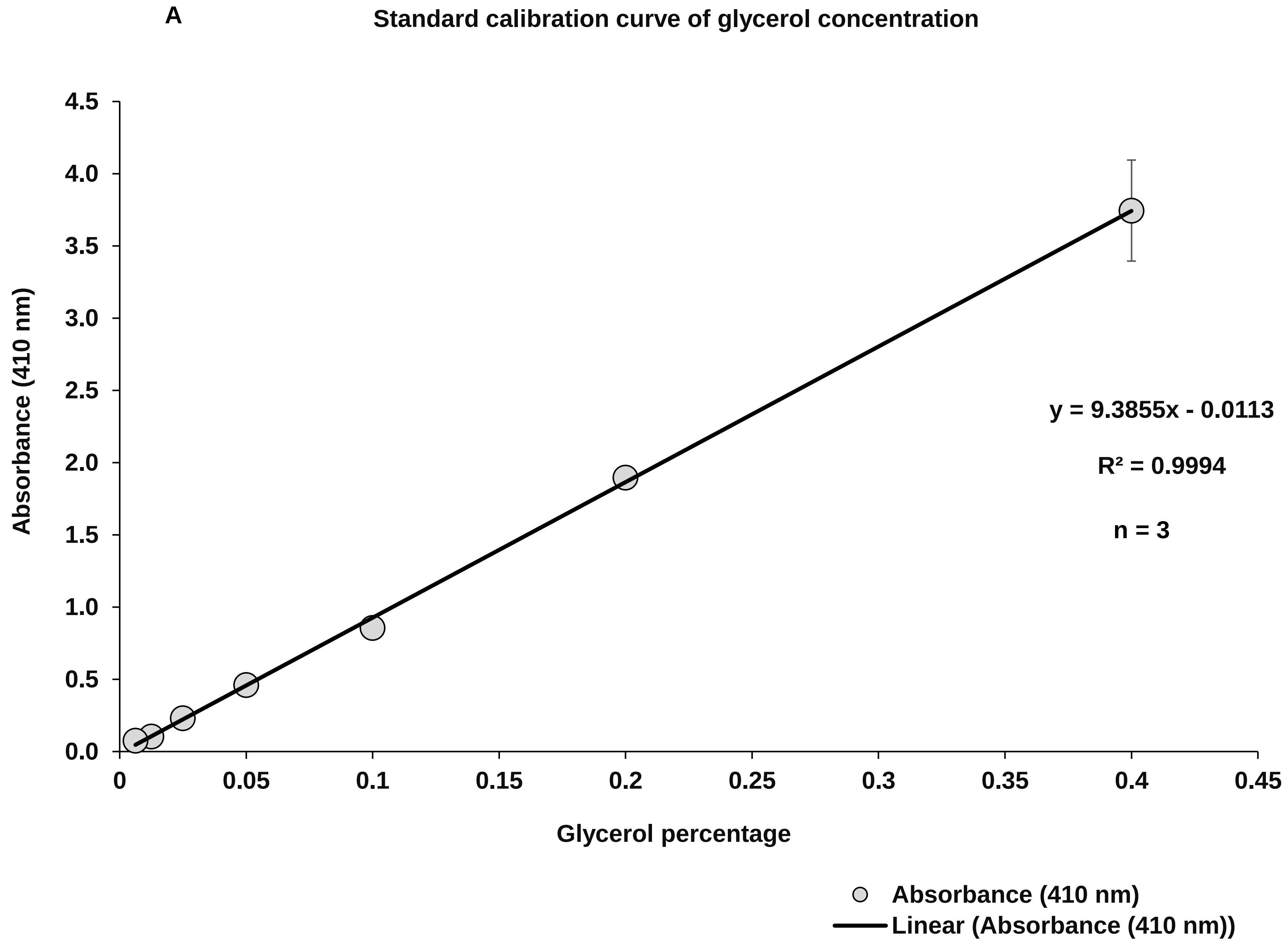

B Glycerol concentrations used for constructing calibration curve

Glycerol Percentage	Absorbance (410 nm)	SD
0.4	3.74	0.35
0.2	1.90	0.07
0.1	0.86	0.02
0.05	0.46	0.04
0.025	0.23	0.04
0.0125	0.10	0.06
0.00625	0.08	0.03

Figure S1. Standard calibration curve for different glycerol concentrations. (**A**) Standard calibration curve. (**B**) Glycerol concentration used for the construction of the calibration curve.

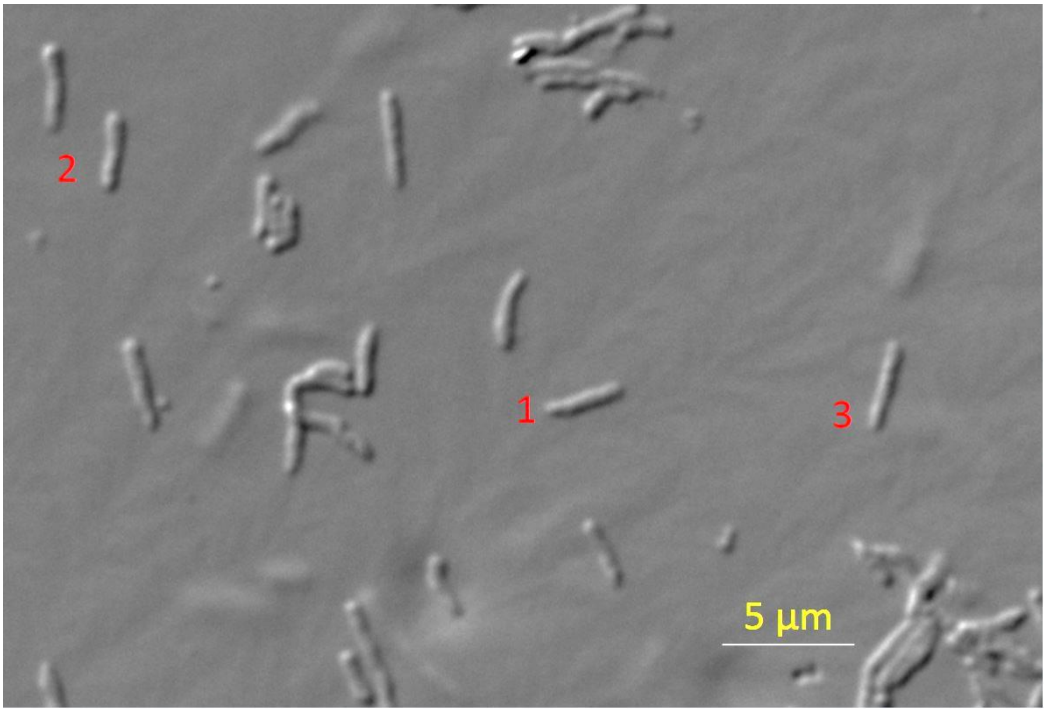

n = 20
N=3

1

n	Measured	Average - measured	error
1	2.92	0.07	0.07
2	2.93	0.06	0.06
3	2.95	0.05	0.05
4	2.95	0.05	0.05
5	2.96	0.04	0.04
6	2.97	0.03	0.03
7	2.97	0.02	0.02
8	2.97	0.02	0.02
9	2.98	0.02	0.02
10	2.98	0.02	0.02
11	2.98	0.01	0.01
12	2.99	0.01	0.01
13	3.01	-0.01	0.01
14	3.02	-0.02	0.02
15	3.04	-0.04	0.04
16	3.04	-0.04	0.04
17	3.07	-0.07	0.07
18	3.07	-0.07	0.07
19	3.07	-0.07	0.07
20	3.08	-0.09	0.09
Average	**2.99**		**0.04**
Relative error	**0.01**		
Percentage error	**1.37**		

2

n	Measured	Average - measured	error
1	2.98	0.06	0.06
2	2.99	0.06	0.06
3	2.99	0.06	0.06
4	3.00	0.05	0.05
5	3.01	0.04	0.04
6	3.02	0.03	0.03
7	3.02	0.03	0.03
8	3.03	0.02	0.02
9	3.04	0.01	0.01
10	3.05	0.00	0.00
11	3.05	0.00	0.02
12	3.06	-0.02	0.02
13	3.07	-0.02	0.03
14	3.08	-0.03	0.04
15	3.08	-0.04	0.04
16	3.08	-0.04	0.05
17	3.10	-0.05	0.05
18	3.10	-0.05	0.06
19	3.11	-0.06	0.07
20	3.12	-0.07	0.00
Average	**3.04**		**0.04**
Relative error	**0.01**		
Percentage error	**1.20**		

3

n	Measured	Average - measured	error
1	3.36	0.08	0.08
2	3.39	0.05	0.05
3	3.40	0.04	0.04
4	3.40	0.04	0.04
5	3.41	0.03	0.03
6	3.41	0.03	0.03
7	3.42	0.02	0.02
8	3.42	0.02	0.02
9	3.43	0.02	0.02
10	3.44	0.00	0.00
11	3.45	-0.01	0.01
12	3.46	-0.02	0.02
13	3.46	-0.02	0.02
14	3.47	-0.03	0.03
15	3.47	-0.03	0.03
16	3.48	-0.04	0.04
17	3.48	-0.04	0.04
18	3.49	-0.05	0.05
19	3.49	-0.05	0.05
20	3.49	-0.05	0.05
Average	**3.44**		**0.03**
Relative error	**0.01**		
Percentage error	**0.9**		

Error = Average of cell size – measured cell size values of individual cell

$$\text{Relative error} = \frac{\text{Average of error}}{\text{Average of cell size measured}}$$

	**1**	**2**	**3**
**Average**	2.99	3.04	3.44
**Relative error**	0.01	0.01	0.01
**Percentage error**	1.37	1.20	0.9

Figure S2. Percentage error in the measurement of three different *Msm* cells.

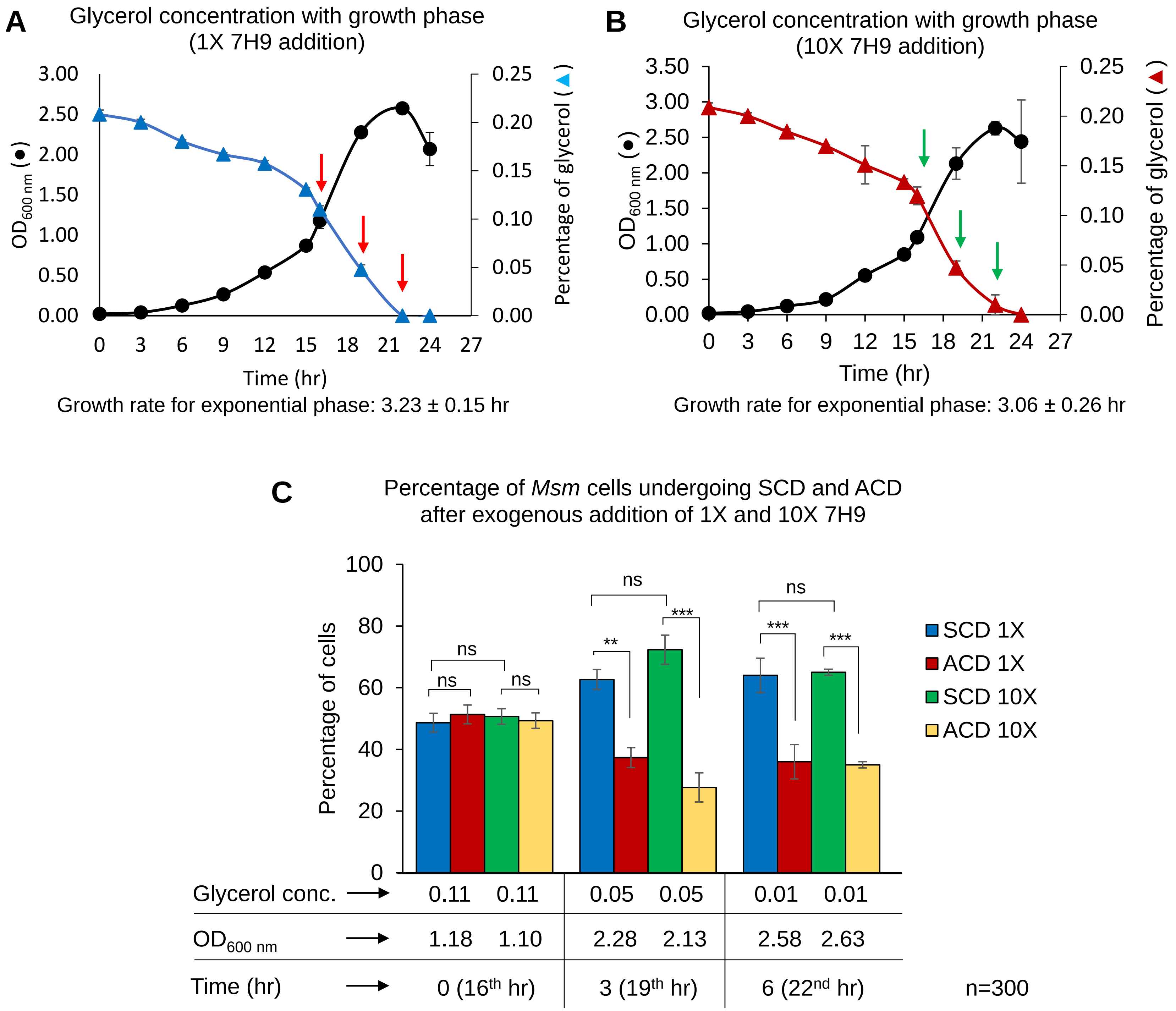

Figure S3. Proportion of the cells undergoing ACD/SCD after addition of 1X Middlebrook 7H9 and 10X Middlebrook 7H9 every 3 hr from 0.1% glycerol concentration. (●) Growth curve of *Msm* cells cultured in Middlebrook 7H9 liquid medium containing 0.2% glycerol and 0.05% Tween 80, with respect to (▲) glycerol concentration after the exogenous addition of (**A**) 0.05% of 1X 7H9 and (**B**) 0.05% of 10X 7H9, at 16th, 19th and 22nd hrs, as indicated by the red and green arrows, respectively. (**C**) OD, glycerol concentration and proportion of SCD (blue and green bar) and ACD (red and yellow bar) after the exogenous addition of 0.05% 1X and 10X 7H9 at 0 hr, 3rd hr and 6th hr. (n = 300. The data shown are for biological triplicates and represented with mean values ± s.d. $p < 0.01$ **, $p < 0.001$ ***, ns – no significance via two-tailed t-test).

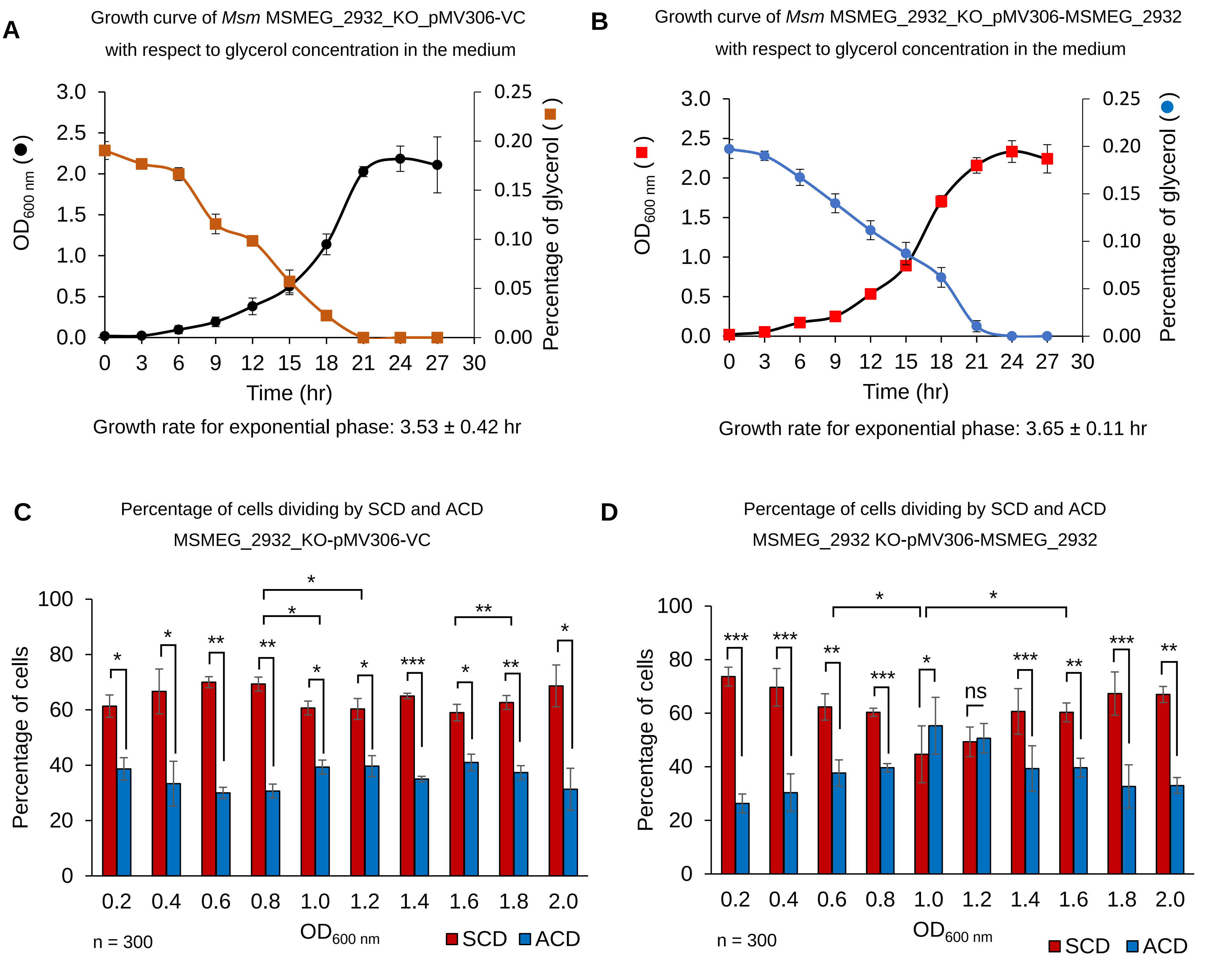

Figure S4. Growth profiles of *MSMEG_2932*_KO_pMV306-VC and *MSMEG_2932*_KO_pMV306-MSMEG_2932, with respect to glycerol levels in the medium, and the proportion of the cells undergoing ACD/SCD. (**A**) Growth curve of *Msm* MSMEG_2932_KO_pMV306-VC (●) with respect to the free glycerol levels in the medium (■). (**B**) Growth curve of *Msm* MSMEG_2932_KO_pMV306-MSMEG_2932 (■) with respect to the free glycerol levels in the medium (●). (**C**) Bar graph for the percentage of cells dividing by SCD (red bar) and ACD (blue bar) of *Msm* MSMEG_2932_KO_pMV306-VC at different $OD_{600\ nm}$ values. (**D**) Bar graph for the percentage of cells dividing by SCD (red bar) and ACD (blue bar) of *Msm* MSMEG_2932_KO_pMV306-MSMEG_2932 at different $OD_{600\ nm}$ values. The data shown are from biological triplicates and represented with mean values ± s.d. ($p < 0.05$*, $p < 0.01$**, $p < 0.001$***, ns – no significance via two-tailed t-test).

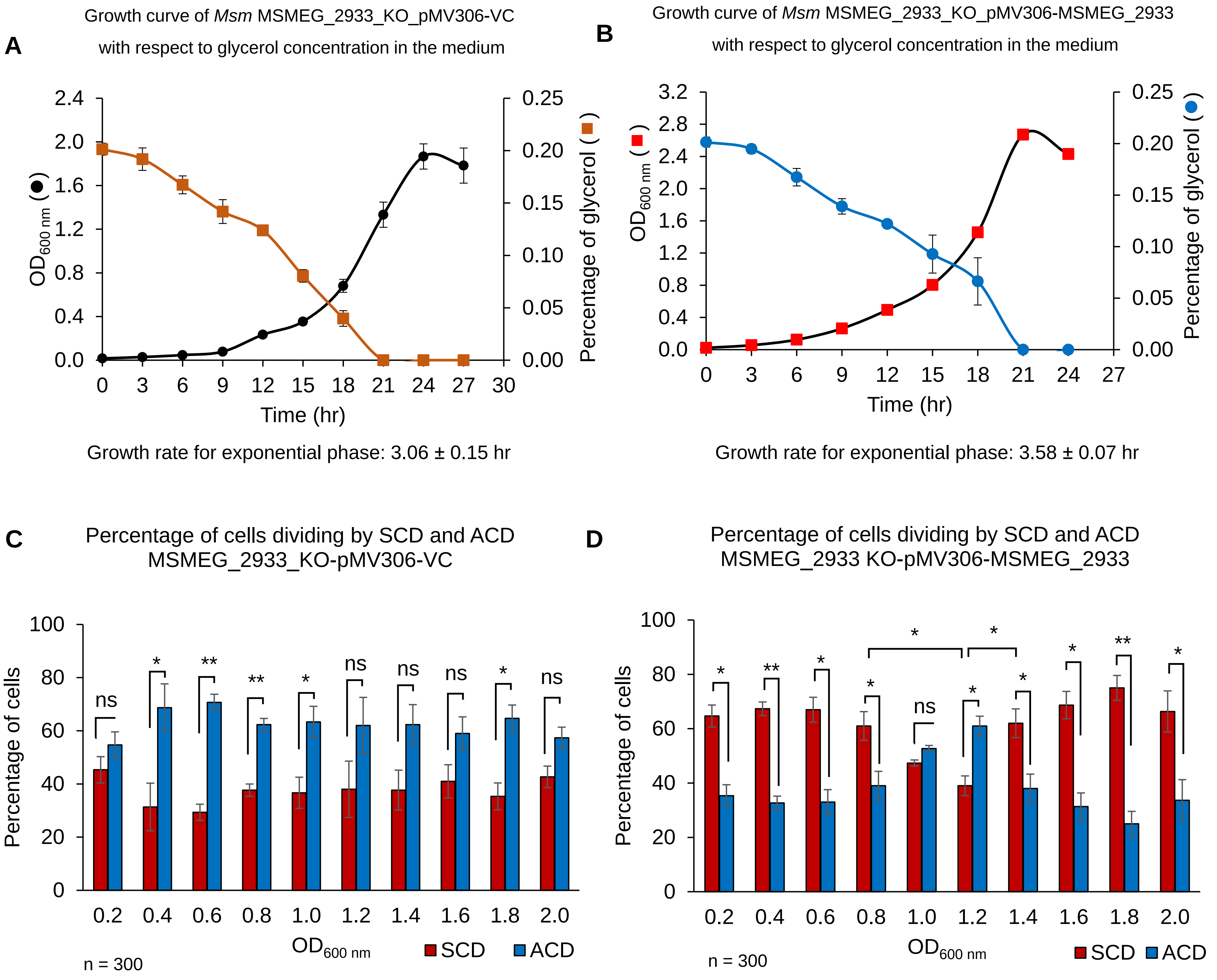

Figure S5. Growth profiles of *MSMEG_2933*_KO_pMV306-VC and *MSMEG_2933*_KO_pMV306-MSMEG_2933, with respect to glycerol levels in the medium, and the proportion of the cells undergoing ACD/SCD. (**A**) Growth curve of *Msm* MSMEG_2933_KO_pMV306-VC (●) with respect to the free glycerol levels in the medium (■). (**B**) Growth curve of *Msm* MSMEG_2933_KO_pMV306-MSMEG_2933 (■) with respect to the free glycerol levels in the medium (●). (**C**) Bar graph for the percentage of cells dividing by SCD (red bar) and ACD (blue bar) of *Msm* MSMEG_2933_KO_pMV306-VC at different $OD_{600\ nm}$ values. (**D**) Bar graph for the percentage of cells dividing by SCD (red bar) and ACD (blue bar) of *Msm* MSMEG_2933_KO_pMV306-MSMEG_2933 at different $OD_{600\ nm}$ values. The data shown are from biological triplicates and represented with mean values ± s.d. ($p < 0.05$*, $p < 0.01$**, ns – no significance via two-tailed t-test).

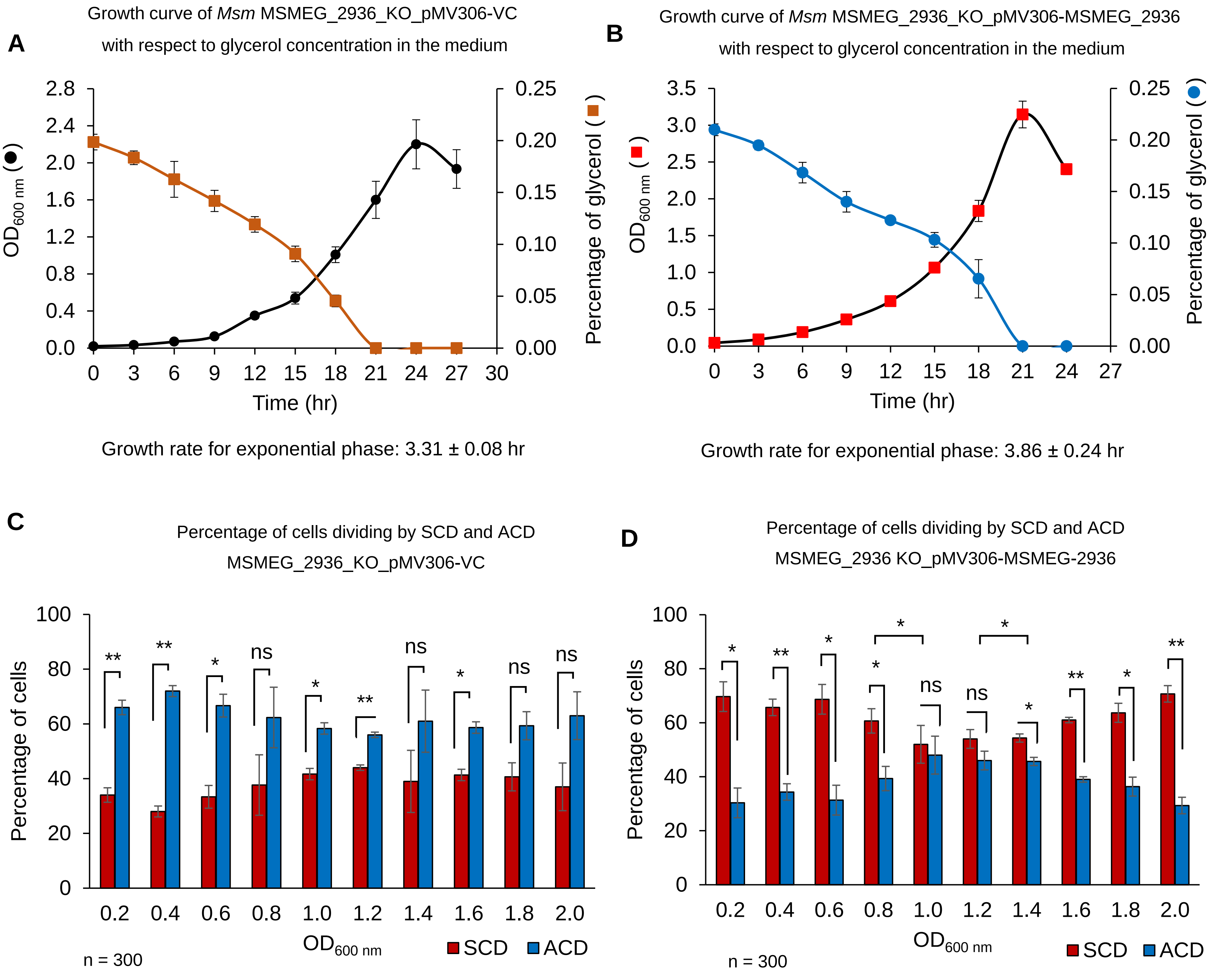

Figure S6. Growth profiles of *MSMEG_2936*_KO_pMV306-VC and *MSMEG_2936*_KO_pMV306-MSMEG_2936, with respect to glycerol levels in the medium, and the proportion of the cells undergoing ACD/SCD. (**A**) Growth curve of *Msm* MSMEG_2936_KO_pMV306-VC (●) with respect to the free glycerol levels in the medium (■). (**B**) Growth curve of *Msm* MSMEG_2936_KO_pMV306-MSMEG_2936 (■) with respect to the free glycerol levels in the medium (●). (**C**) Bar graph for the percentage of cells dividing by SCD (red bar) and ACD (blue bar) of *Msm* MSMEG_2936_KO_pMV306-VC at different $OD_{600\ nm}$ values. (**D**) Bar graph for the percentage of cells dividing by SCD (red bar) and ACD (blue bar) of *Msm* MSMEG_2936_KO_pMV306-MSMEG_2936 at different $OD_{600\ nm}$ values. The data shown are from biological triplicates and represented with mean values ± s.d. ($p < 0.05$*, $p < 0.01$**, ns – no significance via two-tailed t-test).

Scheme S1

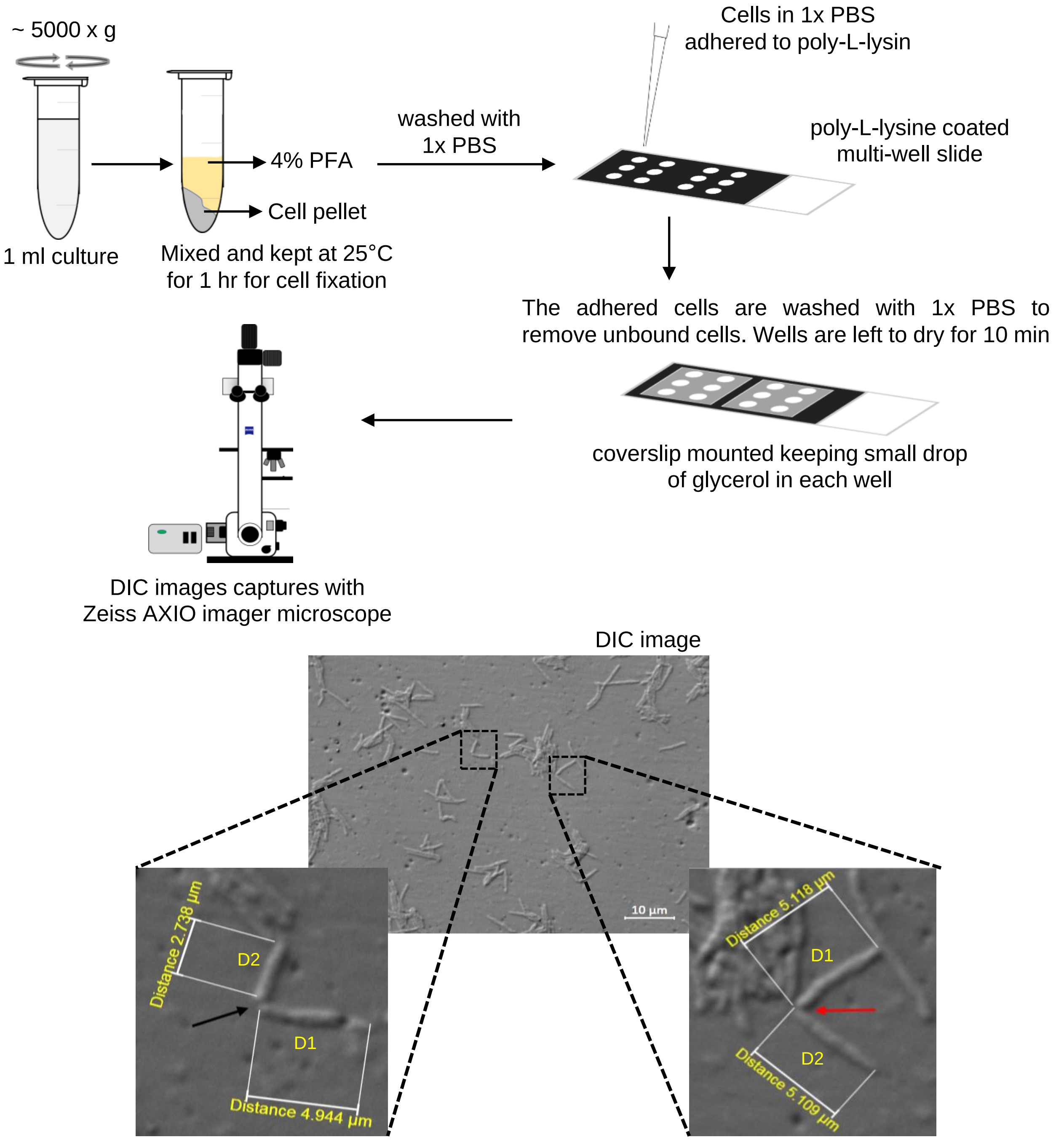

Length D1: 4.944 µm

Length D2: 2.738 µm

D1 + D2 = 7.682; D1 – D2 = 2.206

<u>Percentage deviation from mid-cell site</u>

= 100 x (D1 – D2/ D1+D2)

= 100 x (2.206/7.682) = 28.71

28.71 > 11%; ACD

Length D1: 5.118 µm

Length D2: 5.109 µm

D1 + D2 = 5.118; D1 – D2 = 5.109

<u>Percentage deviation from mid-cell site</u>

= 100 x (D1 – D2/ D1+D2)

= 100 x (0.009/10.227) = 0.09

0.09 < 11%; SCD

Scheme S1. ***Msm* cell harvesting, fixation, DIC imaging and data acquisition for measuring the proportions of cells dividing by ACD and SCD.** One ml of *M. Smegmatis* cells harvested were pellet down at ~5000 x g at 4ºC for 10 min. Cell pellets were fixed in 4% paraformaldehyde (prepared in 1x PBS) for 1 hr of incubation at 25ºC. After washing with 1x PBS, cells were then adhered to poly-L-lysine coated multi-well slides. After drying for ~10 min, the unbound cells were washed-off using 1x PBS in each well. Glycerol (small drop) were kept in each well to mount the coverslip. The cells in each well were captured using Zeiss AXIO imager microscope. DIC images were analysed using ZEN software. Mother cells V-snapped or showing constriction during division. The length of sister-daughter cells in dividing mother cells were measured and percentage deviation from mid-cell site was calculated. The sister-daughter cells were named as D1 and D2, where D1>D2 in cell-length. Percentage deviation of ≥ 11% from mid-cell site were considered as ACD. Similarly, D1 and D2 differing in length by ≤ 11% deviation from mid-cell site, were considered as SCD. Black arrow shows asymmetric constriction during division (ACD) and red arrow shows symmetric constriction during division (SCD).

Scheme S2. Gene knockout strategy and confirmation for *MSMEG_2932*, *MSMEG_2933*, and *MSMEG_2936*

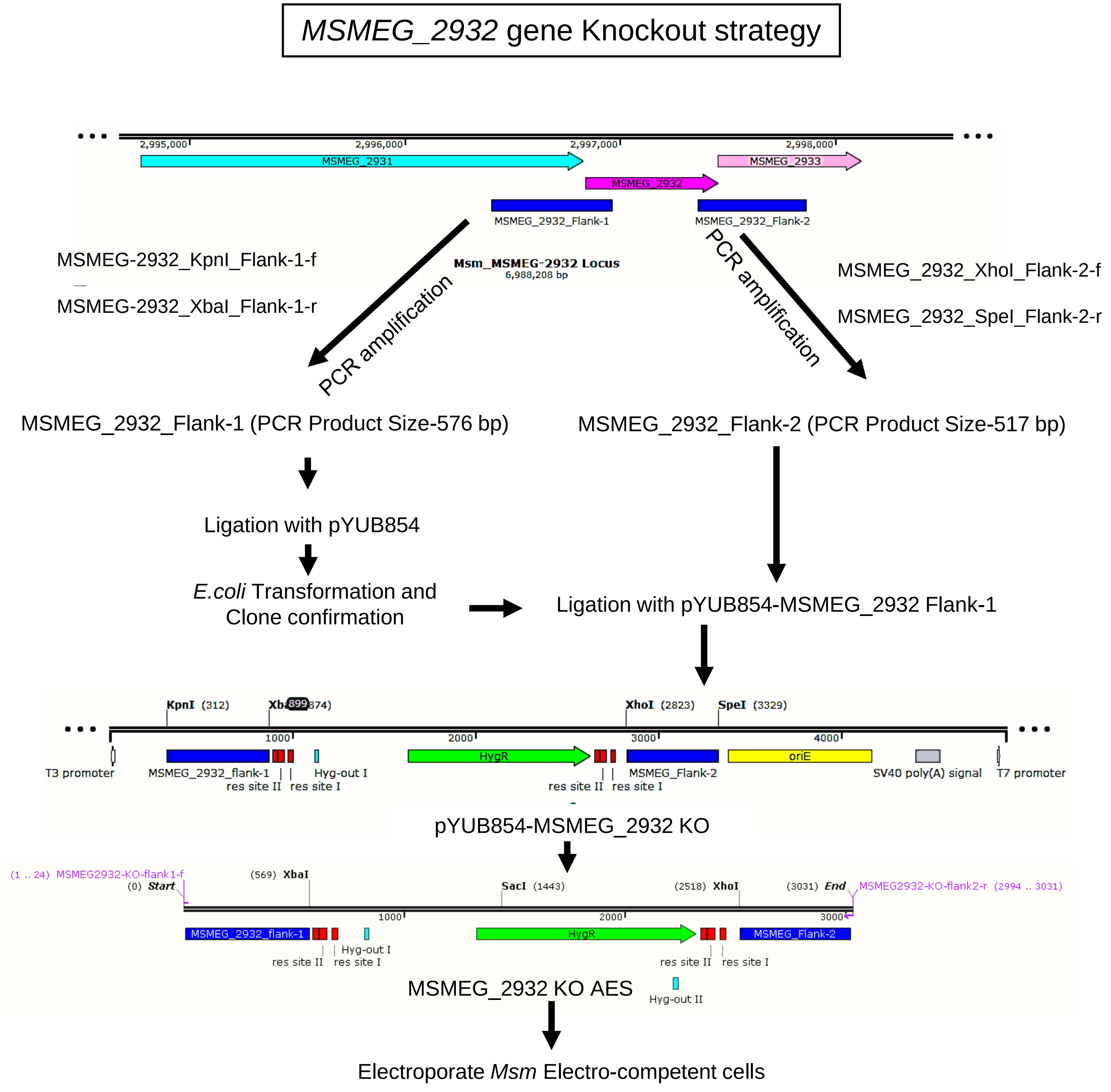

Scheme S2. *MSMEG_2932* gene Knockout strategy. Cartoon depicts the strategy used for the *MSMEG_2932* gene allelic replacement method.

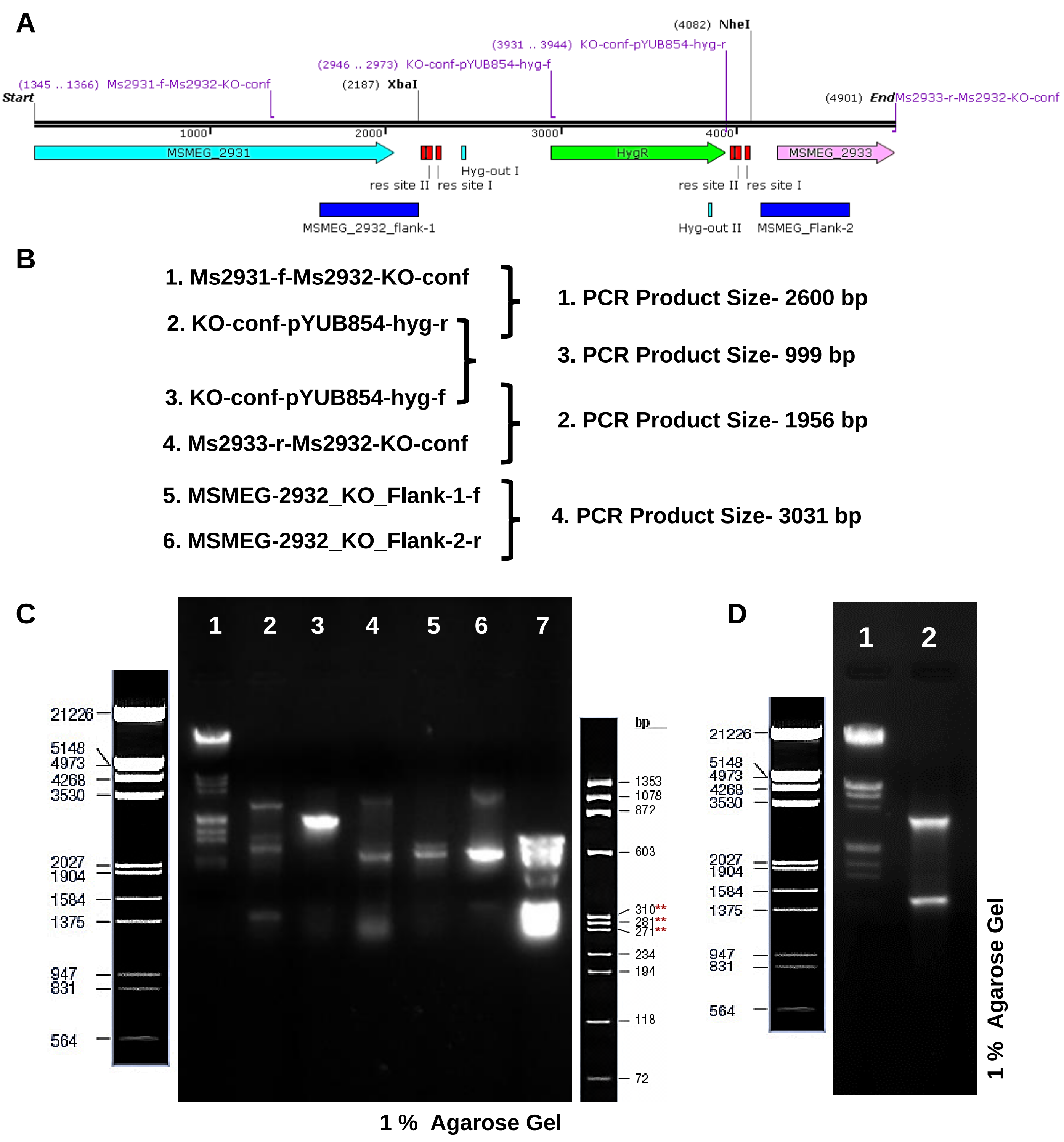

Scheme S3. *Msm MSMEG_2932*-KO clone confirmation using PCR and genomic DNA. (**A**) Cartoon depicts the *MSMEG_2932* gene allelic exchange insert and its PCR Primers for clone confirmation. (**B**) Primers pairs and its respective PCR product details. (**C**) Agarose gel image showing the expected results. Lane-1 Lambda DNA EcoRI/HindIII-Marker-3, Lane 2 PCR Primer set 1, Lane 3 Primer set 2, Lane 4 Primer set 4, Lane 5 Primer set 3, and Lane 6 pYUB854 HygR control, Lane 7 ϕX174 DNA/BsuRI (HaeIII) ,Marker-9. (**D**) Agarose gel image with Primer set 4 Lane-1 Lambda DNA EcoRI/HindIII-Marker-3, Lane 2 PCR Primer set 4.

MSMEG_2933 gene Knockout strategy

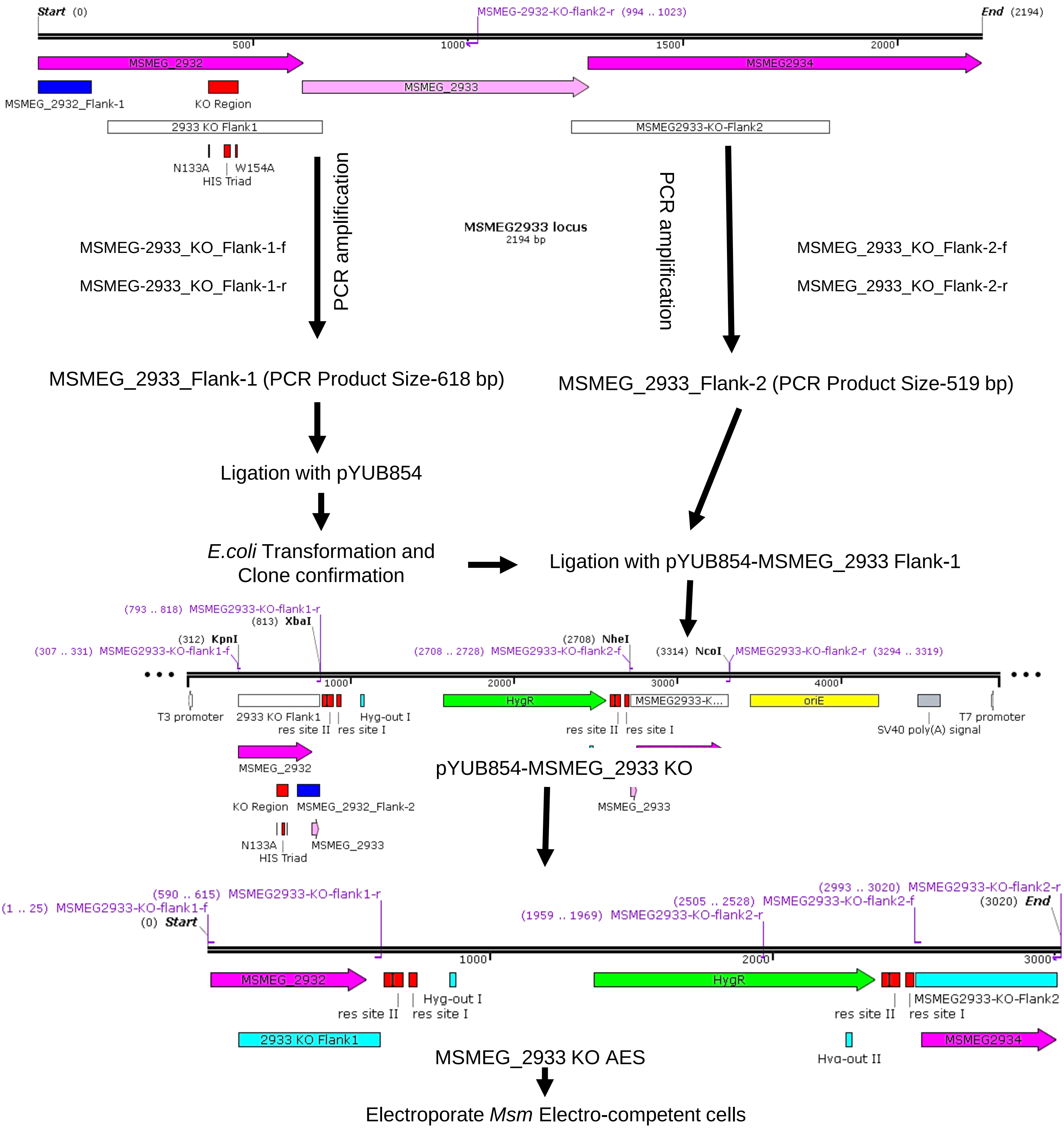

Scheme S4. *MSMEG_2933* gene knockout strategy. Cartoon depicts the strategy used for the *MSMEG_2933* gene allelic replacement method

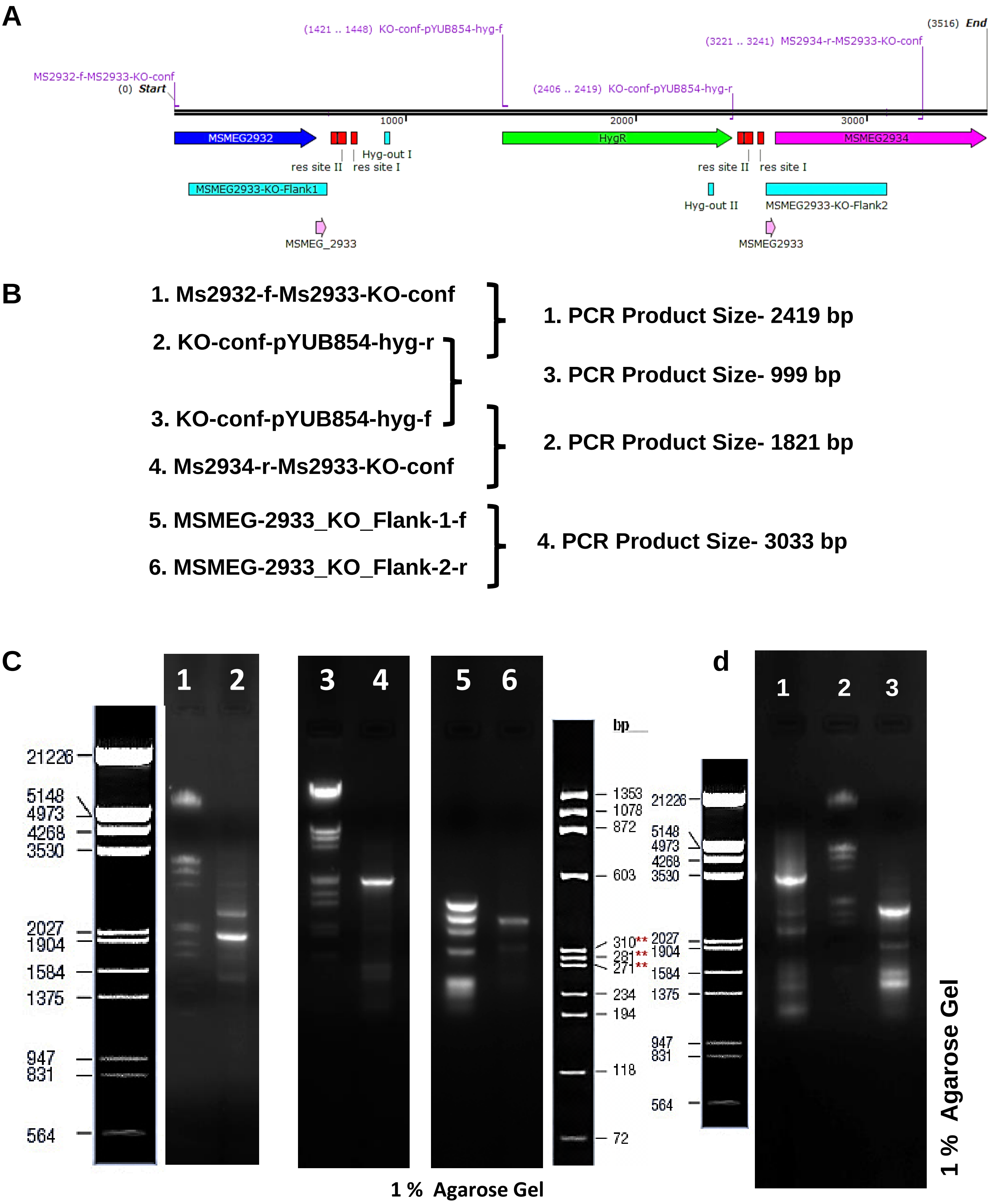

Scheme S5. ***Msm MSMEG_2933*-KO clone confirmation using PCR and genomic DNA.** (**A**) Cartoon depicts the *MSMEG_2933* gene allelic exchange insert and its PCR Primers for clone confirmation. (**B**) Primers pairs and its respective PCR product details. (**C**) Agarose gel image showing the expected results. Lane-1 and 3 Lambda DNA EcoRI/HindIII-Marker-3, Lane 2 PCR Primer set 1, Lane 4 Primer set 2, Lane 6 Primer set 3, Lane 5 ϕX174 DNA/BsuRI (HaeIII) ,Marker-9. (**D**) Agarose gel image for Primer set 4 Lane 1 PCR with *Msm MSMEG_2933* KO gDNA, Lane-2 Lambda DNA EcoRI/HindIII-Marker-3, Lane 3 PCR with *Msm* Wild type gDNA control.

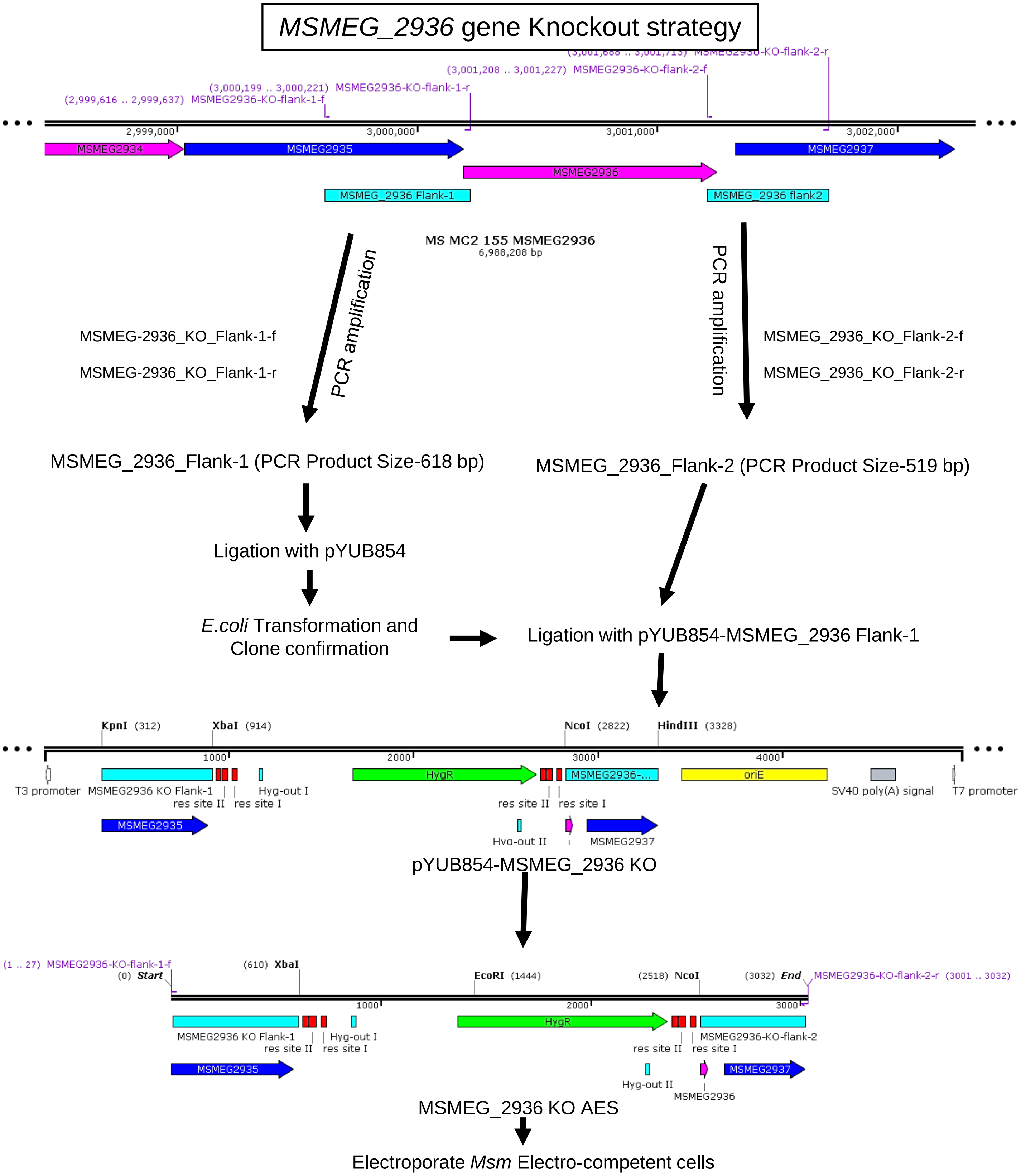

Scheme S6. *MSMEG_2936* gene knockout strategy. Cartoon depicts the strategy used for the *MSMEG_2936* gene allelic replacement method

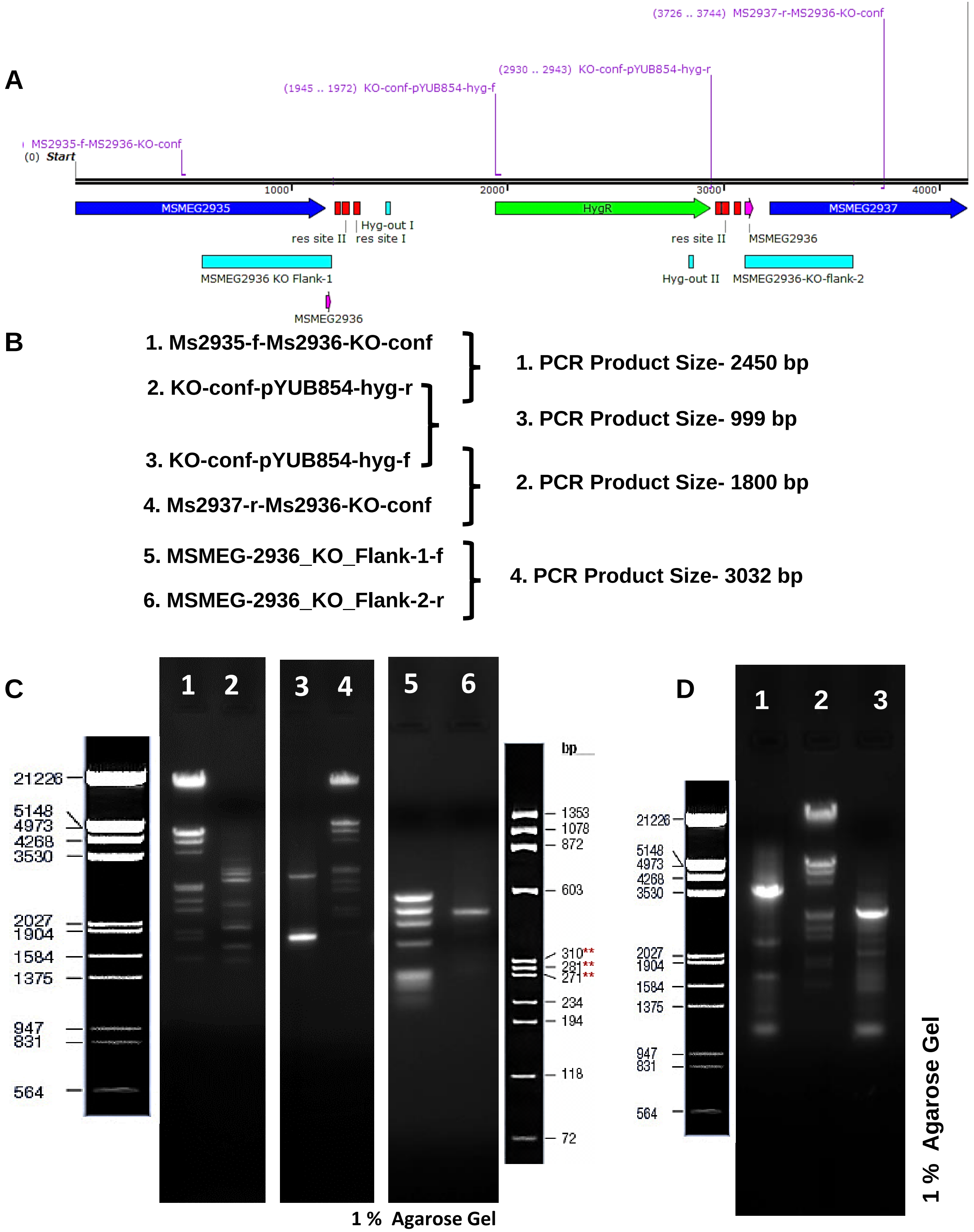

Scheme S7. ***Msm MSMEG_2936 KO*** **clone confirmation using PCR and genomic DNA.** (**A**) Cartoon depicts the *MSMEG_2936* gene allelic exchange insert and its PCR Primers for clone confirmation. (**B**) Primers pairs and its respective PCR product details. (**C**) Agarose gel image showing the expected results. Lane-1 and 4 Lambda DNA EcoRI/HindIII-Marker-3, Lane 2 PCR Primer set 1, Lane 3 Primer set 2, Lane 6 Primer set 3, Lane 5 ϕX174 DNA/BsuRI (HaeIII) ,Marker-9. (**D**) Agarose gel image for Primer set 4 Lane 1 PCR with *Msm MSMEG_2936* KO gDNA, Lane-2 Lambda DNA EcoRI/HindIII-Marker-3, Lane 3 PCR with *Msm* Wild type gDNA control.

Table S1. Bacterial strains and plasmids

Bacterial strains and plasmids	Purpose	Reference
**Bacterial strains**		
Mycobacterium smegmatis mc^{2}155	Experimental system	[1]
Escherichia coli JM109	Cloning host	[2]
Escherichia coli JC10289	Cloning host	[3]
Msm MSMEG_2932 KO	Experimental system	This study
Msm MSMEG_2932 KO-w/o pJV53	Experimental system	This study
Msm MSMEG_2932 KO-w/o pJV53-pMV306	Experimental system, vector control for *Msm MSMEG_2932* KO	This study
Msm MSMEG_2932 KO-w/o pJV53-pMV306-MSMEG_2932	Experimental system, genome integrant complement system for *Msm MSMEG_2932* KO	This study
Msm MSMEG_2933 KO	Experimental system	This study
Msm MSMEG_2933 KO-w/o pJV53	Experimental system	This study
Msm MSMEG_2933 KO-w/o pJV53-pMV306	Experimental system, vector control for *Msm MSMEG_2933* KO	This study
Msm MSMEG_2933 KO-w/o pJV53-pMV306-MSMEG_2933	Experimental system, genome integrant complement system for *Msm MSMEG_2933* KO	This study
Msm MSMEG_2936 KO	Experimental system	This study
Msm MSMEG_2936 KO-w/o pJV53	Experimental system	This study
Msm MSMEG_2936 KO-w/o pJV53-pMV306	Experimental system, vector control for *Msm MSMEG_2936* KO	This study
Msm MSMEG_2936 KO-w/o pJV53-pMV306-MSMEG_2936	Experimental system, genome integrant complement system for *Msm MSMEG_2936* KO	This study

**Plasmids**		
pJV53	Plasmid containing Che9c genes 60-61 under control of acetamidase promoter in pLAM12	[4]
pYUB854	*hyg*^{R} cassette flanked by $\gamma\delta$-res sites and 2 MCSs	[5]
pYUB854-*MSMEG_2932*-KO	560 bp upstream and 500 bp downstream of *MSMEG_2932* gene in pYUB854	This study
pYUB854-*MSMEG_2933*-KO	618 bp upstream and 519 bp downstream of *MSMEG_2932* gene in pYUB854	This study
pYUB854-*MSMEG_2936*-KO	600 bp upstream and 500 bp downstream of *MSMEG_2932* gene in pYUB854	This study
pBS-KS	Cloning/Sequencing vector	[6]
pMV306	Integration vector	[7]
pMV306-*MSMEG_2932*	Integration vector	This study
pMV306-*MSMEG_2933*	Integration vector	This study
pMV306-*MSMEG_2936*	Integration vector	This study

hyg^R is hygromycin resistant

Supplementary References

1. Snapper, S.B.; Melton, R.E.; Mustafa, S.; Kieser, T.; Jacobs, W.R. Jr. Isolation and characterisation of efficient plasmid transformation mutants of *Mycobacterium smegmatis*. *Mol Microbiol* **1990**, *4*, 1911-1919.
2. Yanisch-Perron, C.; Vieira, J.; Messing, J. Improved M13 phage cloning vectors and host strains: nucleotide sequences of the M13mp18 and pUC19 vectors. *Gene* **1985**, *33*, 103-119.

3. Csonka, L.N.; Clark, A.J. Deletions generated by the transposon Tn10 in the *srl recA* region of the *Escherichia coli* K-12 chromosome. *Genetics* **1979**, *93*, 321-343.

4. van Kessel, J.C.; Hatfull, G.F. Recombineering in *Mycobacterium tuberculosis*. *Nature Meth.* **2007**, *4*, 147-152.

5. Bardarov, S.; Bardarov, S. Jr.; Pavelka, M.S. Jr.; Sambandamurthy, V.; Larsen, M.; Tufariello, J.; Chan, J.; Hatfull, G.; Jacobs, W. R. Jr. Specialised transduction: an efficient method for generating marked and unmarked targeted gene disruptions in *Mycobacterium tuberculosis*, *M. bovis* BCG and *M. smegmatis*. *Microbiology* **2002**, *148*, 3007-3017.

6. Alting-Mees, M.A; Short, J.M. pBluescript II: gene mapping vectors. *Nucleic Acids Res*. **1989**, *17*, 9494.

7. Stover, C. K.; de la Cruz, V. F.; Fuerst, T. R.; Burlein, J. E.; Benson, L. A.; Bennett, L. T.; Bansal, G. P.; Young, J. F.; Lee, M. H.; Hartfull, G. F.; Snapper, S. B.; Barletta, R. G.; Jacobs, W. R. Jr. New use of BCG for recombinant vaccines. *Nature* **1991**, 351, 456-460.

Table S2. Oligonucleotide primers used for cDNA synthesis and real time PCR

Name	Sequence
Msm-ThrS-RT-f (MSMEG_2931)	5' atgcaccacctgatcttccg 3'
Msm-ThrS-RT-r (MSMEG_2931)	5' ctcacgcgtggtgtagatgt 3'
Msm-ap4aP-RT-f (MSMEG_2932)	5' aacgtggggctcaatctcg 3'
Msm-ap4aP-RT-r (MSMEG_2932)	5' gttgcctggtctctttcagc 3'
Msm-PI-syn-RT-f (MSMEG_2933)	5' ggcctacgtgaagctttcca 3'
Msm-PI-syn-RT-r (MSMEG_2933)	5' acgaagaacgagacgaccac 3'
Msm-PI-acylT-RT-f (MSMEG_2934)	5' tgcaggtcgacttcttcgg 3'
Msm-PI-acylT-RT-r (MSMEG_2934)	5' gtgtcgagttcgggatagacg 3'
Msm-PI-msylT-RT-f (MSMEG_2935)	5' cacgtgaagttgccggacta 3'
Msm-PI-msylT-RT-r (MSMEG_2935)	5' ggcgatccacttcttgacct 3'
Msm-2936-RT-f	5' tacttcttcgtccaccgcac 3'
Msm-2936-RT-r	5' ccaactcggcgatcattgtc 3'
Msm-16SrRNA-RT-f	5' gcggtaatacgtagggtccg 3'
Msm-16SrRNA-RT-r	5' ttccagtctcccctgcagta 3'

Table S3. Oligonucleotide primers used for the gene replacement of *MSMEG_2932, MSMEG_2933,* and *MSMEG_2936* with res-*hyg*^{R}-res and confirmation

Sl. No.	Primer name	Sequence (5' – 3')	Features
1	MSMEG-2932-KO-flank1-f	ggggtaccatccaccgcgccctgt	PCR amplification of 560 bp upstream to *MSMEG_2932*
2	MSMEG-2932-KO-flank1-r	gctctagagccgcgcttcttcatcggcg	PCR amplification of 560 bp upstream to *MSMEG_2932*
3	MSMEG-2932-KO-flank2-f	ccgctcgagaagagaccaggcaactcttggc	PCR amplification of 500 bp downstream to *MSMEG_2932*
4	MSMEG-2932-KO-flank2-r	ggactagtgcgccttgatgtaggagatcacctgtgagg	PCR amplification of 500 bp downstream to *MSMEG_2932*
5	MSMEG-2933-KO-flank1-f	cggggtaccagcggttgtggacgc	PCR amplification of 618 bp upstream to *MSMEG_2933*
6	MSMEG-2933-KO-flank1-r	tgctctagagaaagcttcacgtaggccgc	PCR amplification of 618 bp upstream to *MSMEG_2933*
7	MSMEG-2933-KO-flank2-f	ctagctagcaccggagacgagcgaacc	PCR amplification of 519 bp downstream to *MSMEG_2933*
8	MSMEG-2933-KO-flank2-r	catgccatggacgaagcggcggtacagc	PCR amplification of 519 bp downstream to *MSMEG_2933*
9	MSMEG-2936-KO-flank1-f	cggggtacccggtacgacgaaccgcgc	PCR amplification of 600 bp upstream to *MSMEG_2936*
10	MSMEG-2936-KO-flank1-r	tgctctagagggtgatgacgatccacaggc	PCR amplification of 600 bp upstream to *MSMEG_2936*
11	MSMEG-2936-KO-flank2-f	catgccatgggcgagatggctcgctcg	PCR amplification of 500 bp upstream to *MSMEG_2936*

12	MSMEG-2936-KO-flank2-r	cccaagcttccacttgtcgatgtggttggtgt	PCR amplification of 500 bp upstream to *MSMEG_2936*
13	Ms2931-f-Ms2932-KO-conf	tacctggagctgtccaccaagg	Clone confirmation for MSMEG_2932 KO
14	Ms2933-r-Ms2932-KO-conf	tcacggttcgctcgtctcc	Clone confirmation for MSMEG_2932 KO
15	MS2932-f-MS2933-KO-conf	gtgaccgatcctgacgagcg	Clone confirmation for MSMEG_2933 KO
16	MS2934-r-MS2933-KO-conf	cgaagaagtcgacctgcacgc	Clone confirmation for MSMEG_2933 KO
17	MS2935-f-MS2936-KO-conf	agatccccaacggtgtcgac	Clone confirmation for MSMEG_2936 KO
18	MS2937-r-MS2936-KO-conf	cgatcttgcgcatgtgcgt	Clone confirmation for MSMEG_2936 KO
19	KO-conf-pYUB854-*hyg*-f	gtgacacaagaatccctgttacttctcg	Confirmation for gene replacement with Hyg^R in MSMEG_2932-KO, MSMEG_2933-KO, and MSMEG_2936-KO
20	KO-conf-pYUB854-*hyg*-r	tcaggcgccggggg	Confirmation for gene replacement with Hyg^R in MSMEG_2932-KO, MSMEG_2933-KO, and MSMEG_2936-KO

Table S4. Coefficient of variation (CV), a measure of the dispersion in the location of the site of constriction from the mid-cell site. (**A**) CV% of population undergoing SCD. (**B**) CV% for population undergoing ACD. (**C**) Geary's statistics w_n, a test of normality for the population undergoing SCD and ACD at OD 0.6, 0.8, 1.0, 1.2, 1.4, 1.6 and 1.8.

**A. CV of the population undergoing SCD**							
**OD**	**P D1**	**P D2**	**SD**	**CV D1**	**CV D2**	**CV% D1**	**CV% D2**
**0.6**	0.55	0.45	0.03	0.06	0.07	6.06	6.09
**0.8**	0.54	0.46	0.03	0.05	0.06	5.24	6.20
**1.0**	0.54	0.46	0.03	0.05	0.06	4.69	5.55
**1.2**	0.54	0.46	0.03	0.05	0.06	4.73	5.55
**1.4**	0.54	0.46	0.03	0.05	0.06	4.88	5.74
**1.6**	0.54	0.46	0.02	0.05	0.05	4.53	5.23
**1.8**	0.54	0.46	0.03	0.05	0.06	4.95	5.82

**B. CV of the population undergoing ACD**							
**OD**	**P D1**	**P D2**	**SD**	**CV D1**	**CV D2**	**CV% D1**	**CV% D2**
**0.6**	0.65	0.35	0.05	0.08	0.16	8.39	15.47
**0.8**	0.66	0.35	0.06	0.09	0.17	8.64	16.49
**1.0**	0.64	0.37	0.06	0.09	0.16	9.22	16.11
**1.2**	0.64	0.37	0.05	0.08	0.15	8.31	14.51
**1.4**	0.64	0.36	0.06	0.09	0.16	8.74	15.56
**1.6**	0.62	0.38	0.04	0.07	0.11	6.91	11.41
**1.8**	0.62	0.38	0.05	0.07	0.12	7.28	11.84

C. W*n* values for the populations dividing by SCD and ACD at different OD

**OD**	**W*n* for SCD**	**W*n* for ACD**
**0.6**	0.86	0.97
**0.8**	0.84	0.96
**1.0**	0.87	0.93
**1.2**	0.82	0.90
**1.4**	0.80	0.93
**1.6**	0.75	0.94
**1.8**	0.77	0.93

Table S5. Oligonucleotide primers used for the generation of genome integrated respective wild type complemented knockout strains of *MSMEG_2932* KO, *MSMEG_2933* KO, and *MSMEG_2936* KO

Sl. No.	Primer name	Sequence (5' – 3')
1	Msm-2932-XbaI-Comp-f	gctctagaacaccaacatgaaggtgccg
2	Msm-2932-HindIII-Comp-r	gaaagcttcacgtaggccgcgcg
3	Msm-2933-XbaI-Comp-f	gctctagatggggctcaatctcggct
4	Msm-2933-EcoRI-Comp-r	cggaattcccagccggccgcatac
5	Msm-2936-XbaI-Comp-f	gctctagagtccaccgctacgactggtc
6	Msm-2936-HindIII-Comp-r	ccaagcttcccggtctggttgctcga

Restriction enzyme recognition sites are underlined
